# Neural mechanisms underlying cognitive inflexibility in obsessive-compulsive disorder: a review

**DOI:** 10.3389/fpsyt.2026.1795436

**Published:** 2026-07-20

**Authors:** Wei Guo, Phoebe Suat-Hong Neo, Neil McNaughton

**Affiliations:** 1 Department of Psychology, University of Otago, Dunedin, New Zealand; 2 Southwest University of Political Science and Law, Chongqing, China

**Keywords:** cognitive inflexibility, cortico-striato-thalamo-cortical network, default mode network, executive function, obsessive-compulsive disorder, salience network

## Abstract

Cognitive inflexibility, a reduced capacity to shift mental set or update behavior when circumstances change, is a significant feature of obsessive-compulsive disorder (OCD). However, while cognitive inflexibility is superficially a coherent entity, it appears to engage a range of distinct cognitive processes. This raises the question of how far deficits in different tasks involve the same core dysfunction and how far the dysfunctions are unique but superficially have similar results. Here we detail the neural basis of OCD deficits across eight tasks that challenge different aspects of cognitive flexibility: set-shifting (Wisconsin Card Sorting Task, Intra/Extra-Dimensional Set Shift), feedback adaptation (Reversal Learning), interference control (Stroop Color and Word Test), response inhibition (Go/No-Go Task and Stop Signal Task), working memory updating (n-back Task), and value-based flexibility (Delay Discounting Task). Cognitive inflexibility in OCD appears linked to functional abnormalities in a largely shared set of core structures (anterior cingulate cortex, caudate nucleus, orbital frontal cortex, and prefrontal cortex) and less shared ‘peripheral’ structures (putamen, thalamus, parietal cortex), based on cross-task convergence of OCD-specific abnormalities. Even the shared core appears to engage multiple interconnected neural networks. The cortico-striato-thalamo-cortical network appears central, and the salience network and the default mode network also contribute with indirect, task-varying effects on peripheral parts of each. Peripheral areas are less consistently involved but appear to contribute to OCD nonetheless. Cognitive inflexibility in OCD appears to involve failure of interaction between multiple networks rather than dysfunction in any single system.

## 1 Introduction

Obsessive-compulsive disorder (OCD) is a heterogeneous condition at both psychological and neural levels. Current evidence continues to implicate cortico-striato-thalamo-cortical network (CSTC) dysfunction, but large-scale brain-wide association studies support this model only partially; whereas task-based studies suggest that distinct neural circuits may be engaged under different cognitive and emotional demands (1). Here we explore the heterogeneity in the context of a limited executive control failure: cognitive inflexibility.

OCD is characterized by the presence of obsessions, compulsions, or both (DSM-5, 2). Obsessions are recurrent and persistent thoughts, urges, or images that are experienced as intrusive and unwanted and that typically cause marked anxiety or distress; the individual attempts to ignore or suppress such thoughts, urges, or images, or to neutralize them with some other thought or action (i.e., by performing a compulsion). Compulsions are repetitive behaviors or mental acts that the individual feels driven to perform in response to an obsession or according to rules that must be applied rigidly; these acts are aimed at preventing or reducing anxiety or distress or preventing some dreaded event or situation, but are not connected in a realistic way with what they are designed to neutralize or prevent, or are clearly excessive. These rigidly applied rules and repeated attempts to neutralize obsessions represent a core behavioral expression of the cognitive inflexibility that characterizes OCD.

In addition to cognitive inflexibility, OCD appears to involve a failure of a range of executive functions (3–7). Core executive functions include inhibition (response and cognitive), working memory updating, and cognitive flexibility (8, 9). Among these, cognitive inflexibility – a reduced capacity to shift mental set or update behavior when circumstances change (10, 11) – appears particularly important in OCD (4, 10). It is manifested in deficits in set-shifting, response inhibition, and goal-directed control (3, 10), compared to various groups, including healthy controls (12, 13), and non-clinical students (14). Cognitive inflexibility underlies the persistence of obsessions and compulsions, contributing to the substantial burden of OCD (11). From a contemporary dimensional and computational perspective, some forms of cognitive inflexibility may be understood in terms of reduced goal-directed, or model-based control over behavior, potentially with a relative shift toward more habitual responding (15–17). In line with this view, Gillan et al. (18) found that deficits in model-based control were most strongly associated with a transdiagnostic dimension of compulsive behavior and intrusive thought, rather than with anxious-depression or social withdrawal. It provides a dimensional and computational perspective on goal-directed deficits relevant to OCD.

Importantly, these deficits persist in remitted patients and are also observed in unaffected first-degree relatives (10, 11, 19), supporting the view that cognitive inflexibility represents a candidate endophenotype (or trait-like marker) of OCD. Their presence has also been associated with increased risk of treatment resistance, although the field has not reached full consensus on the precise contribution of cognitive inflexibility versus other executive deficits (see 1, for a review of treatments in resistant cases). Treatment-resistant OCD (TR-OCD) is typically operationalized as inadequate response to at least one adequate trial of first-line pharmacotherapy and one trial of evidence-based psychotherapy such as cognitive-behavioral therapy with exposure and response prevention, with persistent symptoms remaining (20). Thus, cognitive inflexibility can be usefully framed as a prominent and theoretically important neuropsychological feature of OCD, rather than the sole executive deficit relevant to clinical outcome (10, 11, 21, 22). The precise neural mechanisms of persistent cognitive inflexibility remain unclear – blocking the way to targeted interventions that aim at underlying network dysfunctions (10, 21). Few attempts have systematically integrated evidence across a broad range of cognitive flexibility tasks while simultaneously considering complementary insights from multiple neuroimaging and neuromodulation techniques. The present review addresses this gap by integrating neural findings across eight well-established neuropsychological tasks that probe distinct yet overlapping facets of cognitive flexibility.

It is not clear whether “cognitive flexibility” is a general descriptive term for the effects of multiple processes or the label of a specific process. We will follow Diamond (8) in first assessing “a family of tasks” and, only later, asking if the neural mechanisms we uncover match sufficiently to justify the idea of cognitive flexibility as an overarching process and so cognitive inflexibility as a coherent deficit in OCD. As a starting point, we take cognitive flexibility to be the ability to modify executive function, adapting to internal and external demands (23). Cognitive flexibility builds on working memory and inhibitory control and includes attentional shifting, strategy updating, response to feedback, reversal learning, task switching, exploration, and creative problem-solving (8, 24, 25).

## 2 Methods

This review employs a systematic search strategy to identify relevant literature, ensuring comprehensive coverage, reproducibility and minimizing selection bias, although it is narrative in nature, allowing for an integrative, qualitative synthesis of neural mechanisms of cognitive inflexibility in OCD across heterogeneous tasks and methodologies (26, 27). This hybrid approach leverages the methodological rigor of systematic searches—such as predefined terms, multiple databases, and PRISMA-guided selection—to minimize selection bias, while employing narrative synthesis to facilitate in-depth discussion and interpretation of heterogeneous findings across tasks and networks, where quantitative pooling may not be feasible or appropriate (26–28). Narrative reviews enhanced by systematic methods are suitable to facilitate interpretive discussion of complex, interrelated neural substrates, as recommended for exploratory topics with diverse study designs (e.g., neuroimaging, EEG, and behavioral tasks; 26, 27).

### 2.1 Information sources and search strategy

A comprehensive literature search was conducted across three databases—PubMed/MEDLINE, Web of Science, and Ovid (including APA PsycInfo and Embase)—up to 15 June 2025. The search strategy was designed to identify studies exploring the neural correlates of cognitive flexibility in OCD. Search terms encompassed OCD-related terms (“obsessive-compulsive disorder,” “OCD,” “obsessive”), cognitive flexibility-related terms (“cognitive flexibility,” “cognitive inflexibility,” “set shifting,” “task switching,” “mental flexibility,” and specific tasks such as Wisconsin Card Sorting Test (WCST), Intra/Extra-Dimensional Set Shift (IED), reversal learning, Stroop test, Go/No-Go task, stop signal task, n-back task, delay discounting task, and perseveration), and neural mechanism-related terms (“neural,” “brain,” “circuitry,” “fMRI,” “EEG,” “neuroimaging,” “PET,” “MEG,” “TMS,” “tDCS”). Terms were combined using Boolean operators (AND, OR) and adapted to each database’s syntax (see **Table 1** for full search strings). No language restrictions were applied during the initial search, but only English-language full-text articles were included in the analysis. Non-human studies were excluded. Additionally, reference lists of eligible articles and key reviews were manually screened using back- and forward-snowballing to identify further relevant studies.

**Table 1:** Full search strings for each database.

Database	Search String
PubMed	((“obsessive-compulsive disorder”[MeSH Terms] OR “obsessive-compulsive disorder”[Title/Abstract] OR “OCD”[Title/Abstract] OR “obsessive compulsive”[Title/Abstract]) AND (“cognitive flexibility”[Title/Abstract] OR “cognitive inflexibility”[Title/Abstract] OR “cognitive set shifting”[Title/Abstract] OR “set shifting”[Title/Abstract] OR “cognitive shifting”[Title/Abstract] OR “task switching”[Title/Abstract] OR “mental flexibility”[Title/Abstract] OR “mental set shifting”[Title/Abstract] OR “WCST”[Title/Abstract] OR “Wisconsin Card Sorting Test”[Title/Abstract] OR “IED”[Title/Abstract] OR “intradimensional extradimensional”[Title/Abstract] OR “attentional set-shifting task”[Title/Abstract] OR “reversal learning”[Title/Abstract] OR “Stroop”[Title/Abstract] OR “colour word”[Title/Abstract] OR “Go/No-Go”[Title/Abstract] OR “go no go”[Title/Abstract] OR “stop signal”[Title/Abstract] OR “SST”[Title/Abstract] OR “n-back”[Title/Abstract] OR “n back”[Title/Abstract] OR “delay discounting”[Title/Abstract] OR “intertemporal choice”[Title/Abstract] OR “perseveration”[Title/Abstract]) AND (“neural”[Title/Abstract] OR “brain”[Title/Abstract] OR “circuitry”[Title/Abstract] OR “fMRI”[Title/Abstract] OR “EEG”[Title/Abstract] OR “neuroimaging”[Title/Abstract] OR “PET”[Title/Abstract] OR “MEG”[Title/Abstract] OR “TMS”[Title/Abstract] OR “tDCS”[Title/Abstract]))
Web of Science	TS=(“obsessive-compulsive disorder” OR “OCD” OR “obsessive-compulsive”) AND TS=(“cognitive flexibility” OR “cognitive inflexibility” OR “cognitive set shifting” OR “set shifting” OR “cognitive shifting” OR “task switching” OR “mental flexibility” OR “mental set shifting” OR “WCST” OR “Wisconsin Card Sorting Test” OR “IED” OR “intradimensional extradimensional” OR “attentional set-shifting task” OR “reversal learning” OR “Stroop” OR “colour word” OR “Go/No-Go” OR “go no go” OR “stop signal” OR “SST” OR “n-back” OR “n back” OR “delay discounting” OR “intertemporal choice” OR “perseveration”) AND TS=(“neural” OR “brain” OR “circuitry” OR “fMRI” OR “EEG” OR “neuroimaging” OR “PET” OR “MEG” OR “TMS” OR “tDCS”)
Ovid	(“obsessive-compulsive disorder”.mp. OR “obsessive-compulsive disorder”.ti,ab. OR “OCD”.ti,ab. OR “obsessive compulsive”.ti,ab.) AND (“cognitive flexibility”.ti,ab. OR “cognitive inflexibility”.ti,ab. OR “cognitive set shifting”.ti,ab. OR “set shifting”.ti,ab. OR “cognitive shifting”.ti,ab. OR “task switching”.ti,ab. OR “mental flexibility”.ti,ab. OR “mental set shifting”.ti,ab. OR “WCST”.ti,ab. OR “Wisconsin Card Sorting Test”.ti,ab. OR “IED”.ti,ab. OR “intradimensional extradimensional”.ti,ab. OR “attentional set-shifting task”.ti,ab. OR “reversal learning”.ti,ab. OR “Stroop”.ti,ab. OR “colour word”.ti,ab. OR “color word”.ti,ab. OR “Go/No-Go”.ti,ab. OR “go no go”.ti,ab. OR “stop signal”.ti,ab. OR “SST”.ti,ab. OR “n-back”.ti,ab. OR “n back”.ti,ab. OR “delay discounting”.ti,ab. OR “intertemporal choice”.ti,ab. OR “perseveration”.ti,ab.) AND (“neural”.ti,ab. OR “brain”.ti,ab. OR “circuitry”.ti,ab. OR “fMRI”.ti,ab. OR “EEG”.ti,ab. OR “neuroimaging”.ti,ab. OR “PET”.ti,ab. OR “MEG”.ti,ab. OR “TMS”.ti,ab. OR “tDCS”.ti,ab.)

### 2.2 Eligibility criteria

Included in this review were peer-reviewed articles written in English that either empirically investigated cognitive flexibility in individuals with OCD using neuropsychological tasks or neuroimaging methods, or synthesized such research through reviews or meta-analyses. Duplicate records were identified and removed using Zotero and Rayyan’s duplicate-detection feature by manual verification.

### 2.3 Study selection

**Figure 1:**
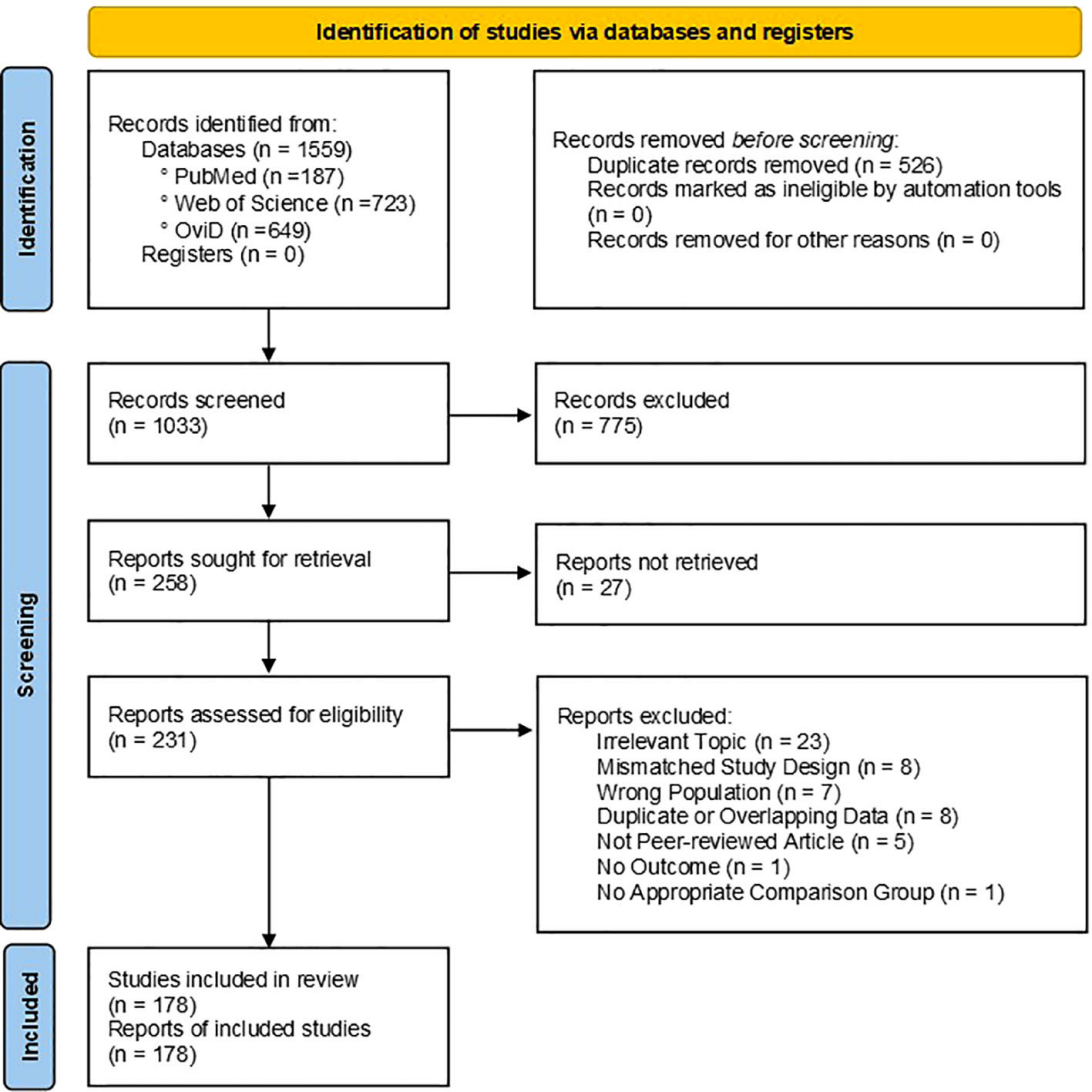
PRISMA flow diagram reproduced from Page et al. (28) under the terms of the Creative Commons CC BY 4.0 license,.

The selection process involved two stages: initial screening of titles and abstracts to identify potentially relevant studies, followed by full-text assessment for eligibility. The overall search and selection process is presented in a flow chart (**Figure 1**) following the PRISMA 2020 format (28), providing an overview of record identification, inclusion, and exclusion at each step. Briefly, a total of 1559 records were identified from the databases (PubMed: 187, Web of Science: 723, Ovid: 649). After removing 526 duplicates, 1033 records were screened. Of these, 775 were excluded based on title and abstract screening, most often due to irrelevance or obvious ineligibility. Full texts of the remaining 258 records were sought, and 231 were retrieved. Upon full-text assessment, 53 reports were excluded for the following reasons: irrelevant topic (n=23), mismatched study design (n=8), wrong population (n=7), duplicate or overlapping data (n=8), not peer-reviewed (n=5), no outcome (n=1), and no appropriate comparison group (n=1). Ultimately, 178 studies were included in the review.

### 2.4 Data extraction and synthesis approach

Data were extracted on study characteristics, task domain, implicated neural regions or circuits, OCD-related behavioral findings, and the direction or nature of reported abnormalities, where available. Studies were first grouped by task domain and then compared across tasks to identify recurrent patterns of OCD-related neural abnormality. Because the included literature was heterogeneous in design, modality, and outcome definition, the synthesis was qualitative rather than meta-analytic. A PRISMA checklist is provided as **Supplementary Table 3**.

## 3 Results

In this section, we detail (**Table 2**) and review (§3.1-3.8) tasks that have been used by those intending to assess cognitive inflexibility in OCD. “Cognitive inflexibility” can have a range of meanings. In this section, we take the specific task to be measuring specific processes and make no assumption that any or all of these processes can be equated with cognitive inflexibility. We leave to §4 an overview of the neural mechanisms involved and of the potential over-arching construct of “cognitive inflexibility”.

**Table 2:** Common brain regions and differential neural basis of deficits in OCD patients (for abbreviations see list in **Supplementary Table 1**).

Task	OCD Task-Common	OCD Task-different
WCST	dACC, Insula ^81^, dCN^81^.	
IED	CN ^84^, vlPFC (BA 10, 11, 47), mPFC (BA 9) ^15,84^.	
RL	OFC, CN, putamen, Insula, dlPFC, dACC, Thalamus ^1,66,85,86,83,65,14,2,26^, vmPFC, lPFC ^1,83,85,66,86,65,14,2^, Parietal Cortex ^66,86,65,14^.	
CWT	ACC ^27,71,^ dlPFC ^58,71^, CN ^75,58^, Thalamus ^28,75^, OFC ^75^, PFC ^75,71,^ Parietal Cortex ^28,43,71^.	Temporal Lobe ^43^, Occipital Cortex ^43,71^.
Go/No-Go	dACC, dlPFC, IFG, PCu, CN, putamen, Thalamus ^5,7,69,37,59,62^, Right OFC ^59,62,69^.	Medial Frontal Gyrus, M1, SMG ^37,62,59,69^, Occipital Cortex ^7,62,69^.
SST	OFC, Right IFG, ACC, CN, putamen ^53,22,32^, Parietal Cortex ^53,22^.	Left pre-SMA, Cerebellum, PCC ^53,22,32^, Occipital Cortex ^32^.
n-back Test	dlPFC, vlPFC, dACC, ACC, Parietal Cortex ^35,36,44,21,73^.	SMA, IPL ^21,35,36,44^, Amygdala ^21^.
DDT	OFC, CN, vmPFC, dlPFC, rACC, dACC, vACC, Thalamus, Right IFG, Insula^13,19,60,17^, rlPFC ^60^.	Cerebellum ^13,60^, Nucleus Accumbens ^19^, STN ^89^.

CWT, (Color and Word Test) – Measures selective attention and interference control through three sub-tasks: reading color names, naming colors of non-word stimuli, and naming the color of incongruently colored words, requiring inhibition of the automatic reading response;

DDT, (Delay Discounting Task) – Evaluates the decline in subjective value with delay. Participants choose between an immediate gain and a larger delayed gain, forming a discounting curve. This curve is assessed using Area Under the Curve (AUC) and the discount constant k. AUC measures the trapezoid area under the curve; the k value models the curve, typically as hyperbolic;

Go/No-Go, Task – Assesses motor response inhibition, with participants responding to “go” cues and withholding responses to “no-go” cues, frequent “go” cues create a prepotent response, requiring inhibitory control on “no-go” trials;

IED, (Intra/Extra-Dimensional Set Shift Task) – Evaluates cognitive flexibility through learning and rule reversal abilities across nine stages, with a focus on extradimensional set-shifting task;

n-back Test, – Assesses cognitive flexibility and adaptive behavior by evaluating the ability to acquire, manipulate, and update information in real-time, essential for adapting to changing environments, through requiring identification of whether the current cue matches the one presented N trials earlier. The difficulty increases with higher values of N;

RL, (Reversal Learning) – Tests the ability to adapt behavior based on changing reward contingencies, requiring subjects to shift response to previously incorrect options when contingencies change;

SST, (Stop Signal Task) – Assesses cognitive flexibility by combining response and stop tasks. Participants must respond to a “go” signal and inhibit response upon a subsequent “stop” signal. The task measures stop signal reaction time (SSRT) as an indicator of inhibitory control;

WCST, (Wisconsin Card Sorting Task) – assesses set-shifting abilities by adapting to changing criteria (color, shape), measuring cognitive flexibility. List of citations: 1. 29 2. 30 3. 31 4. 32 5. 33 6. 34 7. 35 8. 36 9. 37 10. 38 11. 39 12. 40 13. 41 14. 42 15. 22 16. 43 17. 44 18. 45 19. 46 20. 47 21. 48 22. 49 23. 50 24. 51 25. 52 26. 53 27. 54 28. 55 29. 56 30. 57 31. 58 32. 59 33. 60 34. 61 35. 62 36. 63 37. 64 38. 65 39. 66 40. 67 41. 68 42. 69 43. 70 44. 71 45. 72 46. 73 47. 74 48. 75 49. 76 50. 77 51. 78 52. 79 53. 80 54. 81 55. 82 56. 83 57. 84 58. 85 59. 86 60. 87 61. 88 62. 89 63. 90 64. 91 65. 92 66. 93 67. 94 68. 95 69. 96 70. 97 71. 98 72. 99 73. 100 74. 101 75. 102 76. 103 77. 104 78. 105 79. 106 80. 107 81. 108 82. 109 83. 110 84. 13 85. 111 86. 112 87. 113 88. 114 89. 115 90. 116 91. 117.

Supposed direct measures of cognitive flexibility include the Wisconsin Card Sorting Test (WCST), which tests set-shifting by requiring adaptation to changing sorting rules; the Intra/Extra-Dimensional Set Shift (IED) task, which evaluates attentional set-shifting; and Reversal Learning, which measures adaptation to changing reward contingencies. In addition, tasks assessing related executive functions that support flexible behavior are included: the Stroop Color and Word Test measures interference control, suppressing automatic responses to enable task switching; the Go/No-Go and Stop Signal Tasks assess response inhibition, crucial for withholding inappropriate actions to support behavioral adaptation; the n-back task evaluates working memory updating, supporting dynamic information processing for cognitive flexibility; and the Delay Discounting task examines value-based flexibility, reflecting the ability to prioritize long-term goals.

In each subsection we describe the task, discuss the nature of the processes likely being tested, and outline what is known of the neural mechanisms involved. Our overview in §4 will be anchored to the similarities and differences in the brain areas contributing to performance of each task in OCD individuals and so, potentially, underlying OCD deficits.

### 3.1 The Wisconsin card sorting test

*Task:* The WCST assesses set-shifting. Participants must adapt their strategy to changing criteria such as color or shape – a core aspect of cognitive flexibility (118). Tests of poor set shifting in OCD range from the traditional WCST (119) to the more modern Cambridge Neuropsychological Test Automated Battery Intra-Dimensional/Extra-Dimensional Set-Shift Task (CANTAB IDED) test (22). The WCST is still the most commonly used.

*Neurology:* Ventromedial prefrontal cortex (PFC) lesions impair adaptation in the WCST (34), as with earlier reports of frontal lobe deficits in switching between solution modes (82). The PFC’s involvement in behavioral flexibility during rule shifting is also evidenced by fMRI (72, 83, 84).

*OCD deficits:* Various studies show reduced cognitive flexibility in OCD patients compared to control and generalized anxiety groups, regardless of medication use or comorbidity (120, 121). When compared to GAD and social anxiety disorder groups, OCD patients exhibit the least cognitive flexibility (122). However, Wang et al. (123) found no significant difference in set-shifting abilities between OCD patients and healthy controls, furthermore, the rate of perseverative errors on the WCST did not correlate with the severity of checking symptoms, highlighting the intricate nature of cognitive flexibility and its variable relationship with different OCD types.

*Neural basis of deficits:* Research using the WCST has revealed distinct patterns in functional connectivity in OCD patients compared to healthy controls. Specifically, increased connectivity between the dorsal caudate, dorsal anterior cingulate cortex, and anterior insula in OCD patients was linked to decreased cognitive flexibility, while the opposite was true for healthy controls (108).

### 3.2 The intra/extra-dimensional set shift task

*Task:* The IED task is a comprehensive test for evaluating cognitive flexibility through learning and rule reversal abilities. This task, as described by Sahakian & Owen (124), consists of nine stages, primarily focusing on the first seven stages where the participant’s response to a specific stimulus dimension is evaluated. The extradimensional set-shifting task (EDS), the eighth stage, is crucial for assessing cognitive flexibility, as it measures the ability to shift attention from a previously correct stimulus to a different dimension.

*Neurology:* The IED task activated the left anterior prefrontal cortex and the right dorsolateral prefrontal cortex (BA 10 and 9/46) (95). More total errors on the IED task were associated with decreased grey matter probability in the ventral tegmental area, medio-prefrontal cortex, and periaqueductal grey, while increased pre-ED errors were associated with significantly decreased white matter in the anterior cingulate cortex, bilateral insula, and nucleus accumbens septi (107). Deep brain stimulation of the anteromedial subthalamic nucleus substantially improved cognitive flexibility, evidenced by reduced EDS errors (109).

*OCD deficits:* Results with OCD are inconsistent. Chamberlain et al. (22) found significant deficits in extradimensional shifting but Ren et al. (125) did not. Kim et al. (126) found similar cognitive flexibility and visual processing to typically-developing controls in OCD, but deficits in GAD.

*Neural basis of deficits:* Chamberlain et al. (22) found dysfunction in specific areas of the fronto-striatal pathways. Their study, which analyzed data from 335 OCD patients and 311 controls, found medium to large effect size deficits in cognitive flexibility related to fronto-striatal pathway dysfunction. This dysfunction involved the ventrolateral (Brodmann areas 10, 11, and 47) and medial (Brodmann area 9) prefrontal cortex, as well as the caudate nucleus.

### 3.3 Reversal learning

*Task:* In reversal learning, after learning the correct one of two alternatives, participants must shift to what was previously the incorrect option – adapting behavior to changed reward patterns (choice contingencies). The choice stimuli do not change, only which stimulus is correct.

*Neurology:* Impaired recruitment of the orbitofrontal cortex (OFC, 29, 42, 92, 93), CN and putamen (29) have been linked to difficulties in adapting to reversal tasks. Patients suffering from unilateral hippocampal sclerosis exhibited habitual and inflexible behavior (114), consistent with a more general role of the hippocampus in behavioral inhibition (127).

*OCD deficits:* Valerius et al. (110) found prolonged reaction times, which correlated with the severity of compulsions. Veale et al. (111) found deficits in both acquiring and maintaining cognitive sets. Importantly, OCD patients were also impaired in simple discrimination learning with more patients failing at each stage as the task became more difficult.

*Neural basis of deficits:* Multiple interconnected brain regions, particularly the OFC-striatal loop and PFC, are implicated. There is reduced activity during reward processing in the reversal learning task in the OFC-striatal loop (93, 112) and the right medial and lateral OFC and right caudate (92). There is reduced activity during affective switching in the OFC, dlPFC, bilateral anterior PFC, and anterior insula (92, 93). The dlPFC exhibits robust hypoactivity in OCD patients during reversal learning (29, 92, 94). Both OCD patients and their unaffected relatives showed reduced activation bilaterally in the lateral OFC, lateral PFC, and parietal cortex during reversal learning (42).

The failure to differentiate between safe and threatening stimuli during reversal learning is associated with the absence of ventromedial prefrontal cortex (vmPFC) safety signaling and increased connectivity between the vmPFC and salience network regions, including the dorsal anterior cingulate, insula, and thalamus (30).

Endrass et al. (53) found that OCD patients have impaired feedback monitoring during reversal learning tasks. They also found OCD patients exhibited reduced feedback-related negativity (FRN) amplitudes for exploration negative feedback during a reversal learning task, suggesting attenuated monitoring of feedback, which potentially contributes to deficits in adaptive behavior reflected in obsessive thoughts and actions.

### 3.4 The Stroop color and word test

*Task:* The CWT measures executive functions such as selective attention and interference control (128, 129). The test consists of three sub-tasks. First, participants are required to read within a fixed time as many words that denote colors as possible printed in black ink. Second, they are required to name the colors of displayed non-word stimuli. The final interference sub-task involves incongruently colored color-words (e.g., the word “red” printed in blue ink). Participants should name the color of the word while inhibiting the automatic tendency to read the word itself. Response time in the last condition is slowed due to the competing processing of semantic and visual content.

*Neurology: The interaction of* ACC (error detection) and the dlPFC (management of interference) is important for the CWT (52, 81). The ACC detects cognitive conflicts particularly outcome errors (38, 69) and signals the dlPFC to increase cognitive control (52).

*OCD deficits:* Results are varied. Yazdi-Ravandi et al (130) found slower reaction times and higher error rates in color-word test trials. Similarly, Martínez-Esparza, et al. (121) reported greater interference. In contrast, others found no significant differences between OCD patients and the healthy groups in the CWT test (120, 131–133).

*Neural basis of deficits:* The ACC and dlPFC have shown decreased functional coupling to temporo-limbic areas (55). Impairments in cognitive flexibility and inhibition in OCD patients during the CWT are correlated with dysfunction in frontal (102) and temporal brain regions (70). Marked difficulties in inhibitory control during the CWT in OCD patients, especially those with reactive symptoms, are associated with deficits in the ACC and related PFC (54). There also appears to be increased subcortical functional coupling from bilateral striatal and thalamic regions toward cortical areas of the parietal, occipital, and temporal lobes in OCD patients (55).

### 3.5 The Go/No-Go Task

*Task:* The Go/No-Go task assesses inhibition of a prepotent motor response (134, 135). Two stimuli, frequent “go” and less frequent “no-go”, are interspersed. Participants should respond to the “go” cue but not the “no-go” cue (11).

*Neurology:* The Go/No-Go task involves several brain regions in the inhibition process: right prefrontal cortex, including the posterior part of the inferior frontal gyrus (IFG) and the adjacent part of the middle frontal gyrus (MFG). The ACC is commonly activated, though its role is attributed more to selective, executive attention and performance monitoring, particularly during failed inhibition trials (39). In contrast to the more lateral regions of the lateral OFC, the rostro-medial regions of the lateral OFC seem to be involved in behavioral inhibition more generally (79).

*OCD deficits:* In the Go/No-Go task, individuals with OCD can show significant difficulties with response inhibition – with higher rates of both commission and omission errors compared to controls (121, 136, 137; but see 120 on omission errors). Commission errors, where subject responds to “no-go” cues, indicate a difficulty in suppressing actions. Omission errors, where subject fails to respond to “go” cues, suggest challenges in initiating responses. These errors reflect a broader difficulty in managing motor and cognitive control necessary for correctly performing tasks that demand precise behavioral inhibition. Patients with GAD exhibited better motor response inhibition scores than those with OCD and Social Anxiety Disorder but with only moderate effect sizes (122).

*Neural basis of deficits - cortical:* Error-related brain activity is negatively associated with OCD scale scores in the dorsal dACC, left thalamus, and bilateral occipital cortex (35). OCD patients show under-activation in the aOFC, dlPFC (89), inferior and medial frontal gyri, with symptom severity in OCD inversely correlated with activation in the right orbitofrontal and anterior cingulate gyri. Greater activation in the right dlPFC and right supramarginal gyrus in OCD patients compared to healthy controls is observed during the Go/No-Go task (64). Pediatric OCD patients show significantly increased amplitude of activity in the primary motor cortex (M1) in response to the visual cue presented during the Go condition compared to typically developing controls (TDC). Additionally, OCD patients exhibit significantly reduced amplitude in the precuneus (PCu) following successful stopping to No-Go cues compared to TDC (86).

*Neural basis of deficits - subcortical:* The CN and putamen (89), are frequently reported as abnormal in OCD. Becker et al. (35) found negative associations between error-related activity in these regions and OCD symptom severity. Roth et al. (96) found that symptom severity in OCD was positively correlated with activation in the thalamus and posterior cortical areas during response inhibition in Go/No-Go task. Becker et al. (35) similarly reported negative associations between error-related activity in the left thalamus and OCD symptom severity.

*Neural basis of deficits - Event-related potentials (ERPs)*: ERP studies have demonstrated that OCD patients have functional abnormalities in multiple brain regions in the Go/No-Go task, including the dACC, prefrontal cortex, CN, putamen, thalamus, and various cortical areas. There is atypical topography of the No-Go N2 component, with a more posteriorly distributed N2 amplitude and an absence of the usual strong frontally maximal topography, along with longer N1 latencies and smaller P2 amplitude to No-Go stimuli compared to healthy controls (138). The usual increase in N2 latency with increasing inhibitory difficulty is absent (138); as is the usual differences in ERN and CRN amplitudes, and affective priming is decreased (33).

### 3.6 The Stop Signal Task

*Task:* The SST combines response and stop tasks to assess cognitive flexibility (139). Initially, a fixation point “+” appears in the center of the screen, promptly followed by a left or right arrow. Subjects are instructed to make a swift, appropriate (left/right) response. If a stop signal is then presented, they must stop making the response. The “stop signal delay” (SSD) between the two stimuli varies – being increased after correct and decreased after incorrect responses – so performance tends to track 50% correct stopping. The estimated stop signal reaction time (SSRT) assesses inhibitory action control.

**Figure 2:**
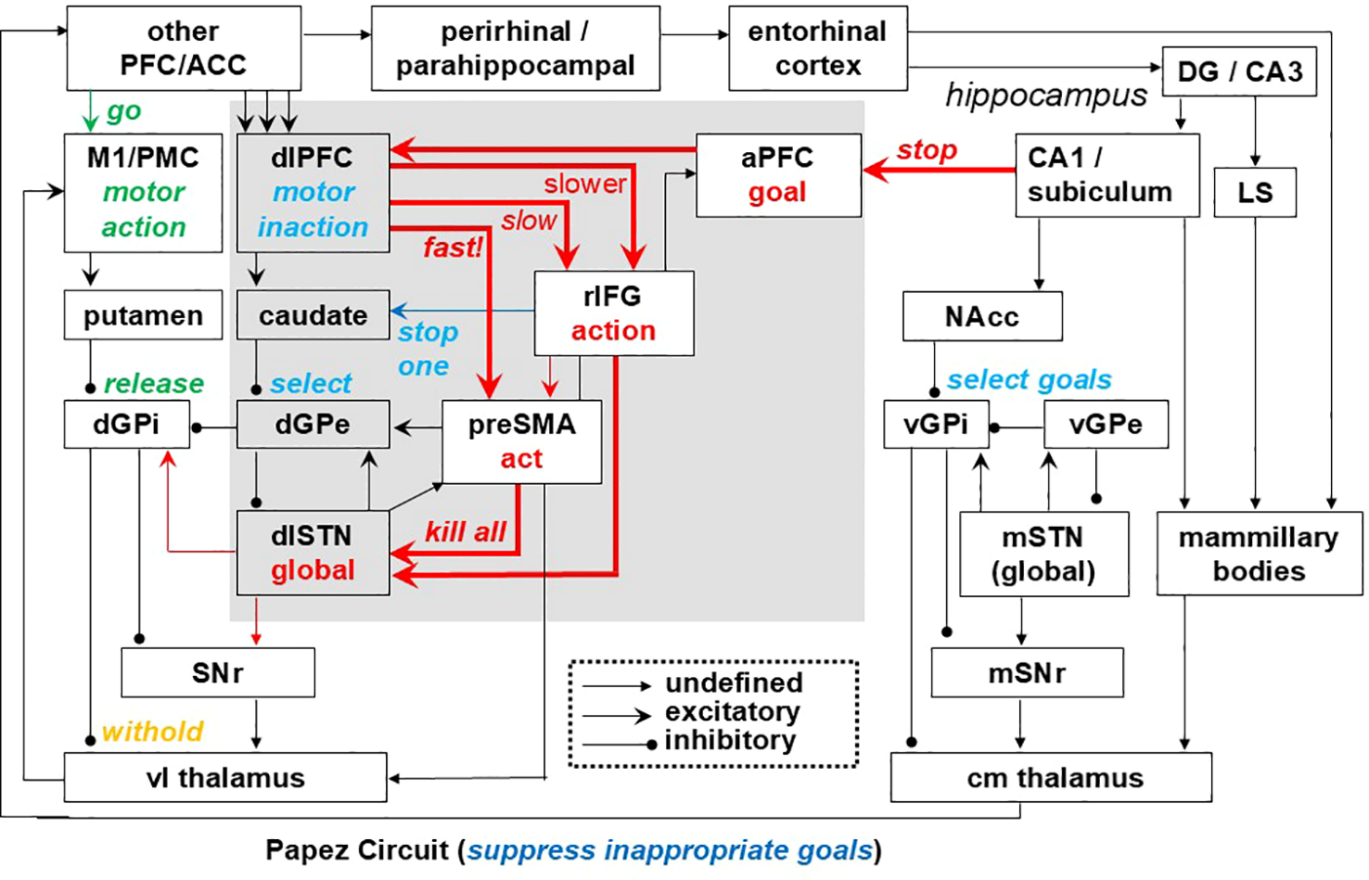
The key circuits involving the interaction of going and stopping in frontal circuits in the SST and Go/No-Go tasks. This is simplified in that each element of the diagram stands for a set of parallel circuits. “Going” (green/blue, left hand side) is intended to include choices where selected (blue) action alternatives are inhibited and where the executed action is released from inhibition. Action choice is thus often inhibition of inhibition. Thus, available stimuli can ‘prime’ a range of actions and plans, with execution requiring only release. “Stopping” (red, grey background) includes kill switch, selective stopping, action choice, and goal inhibition. Goal inhibition is generated as one (“stop”) output from the Goal Inhibition System (hippocampus) that is distinct from its (right hand side) arousal and attention outputs and from its role in goal section. Immediate goal inhibition/stopping (red arrow) of the competing outputs directed towards positive and negative goals (attractors/repulsors) allows sophisticated forms of conflict resolution to occur without interference. Lowercase abbreviations: a = anterior c = central, d=dorsal, e = pars externalis, i = pars internalis, l = lateral, m = medial, r = right, v = ventral. Uppercase abbreviations: ACC = anterior cingulate cortex, DG = dentate gyrus, GP = globus pallidus, IFG = inferior frontal gyrus, LS = lateral septum, M1 = primary motor cortex, PFC = prefrontal cortex, PMC = premotor cortex, SNr = substantia nigra pars reticulata, SMA = supplementary motor area, STN = subthalamic nucleus. Figure and adapted legend from McNaughton & Gray (127) with permission of the authors and via contract.

*Neurology:* In summary, the studies (e.g., 31, 32, 37, 40, 45, 56, 57, 66–68, 73, 88, 97, 99, 104, 105, 113, 116, 117, 140) collectively reveal a complex network of brain regions, including the pre-SMA, IFG, parietal cortex, basal ganglia, other frontal cortex, as well as visual areas and the cerebellum, which cooperate during response inhibition in the SST (see **Figure 2**). Integrating these findings provides a comprehensive description of the neural mechanisms underlying response inhibition and its variation with task demands. It should be noted that the other tasks reviewed here are likely to have similarly complexity in terms of parallel circuit organization.

*OCD deficits:* There is clear impairment of SST performance in OCD patients and their first-degree relatives. Menzies et al. (80) found moderate yet significant impairments in SSRT performance, with delayed response inhibition in OCD patients and their unaffected first-degree relatives compared to healthy controls. De Wit, et al. (49) also observed greater stop-signal reaction times among OCD patients relative to healthy control subjects. Moreover, Berlin and Lee (141) found that SSRT predicted compulsion severity, suggesting that compulsions in OCD might primarily stem from failed response inhibition. This suggests that further research is needed to clarify the specific mechanisms between response inhibition and the various symptoms of OCD. Importantly for any links with anxiety, SSRT is not changed by anxiolytic drugs even when EEG activity linked to stop-go conflict is reduced (142).

*Neural basis of deficits:* OCD patients and their unaffected first-degree relatives had reduced grey matter in the orbitofrontal and right inferior frontal gyrus, as well as increased grey matter in the cingulate, parietal, and striatal regions (80). Heightened activity in the left presupplementary motor area during successful inhibition in OCD patients and their siblings compared to healthy controls, suggests a compensatory mechanism for their impaired inhibitory control, whereas OCD patients exhibited decreased activity in the right inferior parietal cortex and inferior frontal gyrus relative to both healthy control subjects and their own siblings, suggesting a potential genetic component to this disorder (49). Patients with OCD and their first-degree relatives had abnormally reduced functional connectivity during the SST between frontal and posterior brain regions. These regions included the frontal cortex, occipital cortex, and cerebellum (59). Collectively, these studies indicate that the orbitofrontal cortex, ACC, PCC, parietal cortex, CN, putamen, left presupplementary motor area, inferior frontal gyrus, occipital cortex, and cerebellum may be involved in the neural deficits during the SST observed in OCD.

### 3.7 The n-back test

*The task:* The n-back test is designed to evaluate working memory and cognitive updating. Subjects are presented with a continuous sequence of cues, generally letters or numbers, and must identify whether the current cue matches the one presented n trials earlier. As the value of n increases, so does the difficulty of the task (11, 48). Although traditionally considered a measure of working memory, it now seems that updating is also important (11).

*Neurology:* Activation during the n-back task involves a cortical network including the bilateral PFC, bilateral premotor areas (PreMA), SMA, and bilateral parietal cortex (75). Difficulty level increased signal intensity in anterior regions, including the dorsolateral PFC, premotor cortex, and SMA (103). There is task-related activation in the superior frontal sulcus, dlPFC, bilateral ventrolateral prefrontal cortex, inferior frontal gyrus, premotor cortex, precentral gyrus in the primary motor cortex, inferior parietal lobe, intraparietal sulcus, and visual association areas including the lingual and fusiform gyri (51).

The parietal and occipital cortex also play roles in the n-back task, with pre-training activations in the parietal-occipital lobe (including Superior Parietal Lobule, Inferior Parietal Lobule, the superior, middle, and inferior occipital gyri), bilateral PFC (including the superior frontal gyrus, middle frontal gyrus, and inferior frontal gyrus), and left cerebellum, and post-training activations in the left parietal-occipital lobe (including the superior parietal lobule, inferior parietal lobule, and the middle and inferior occipital gyri) and left cerebellum (50). Additionally, significant bilateral activation clusters during the task are found in the parietal cortex, visual cortex, PFC, insular cortex and ACC, with right parietal cortex activation inversely correlated with intelligence (106).

The ACC and SMA are crucial for the n-back task, with higher activation in bilateral superior/middle/inferior frontal, inferior/superior parietal, medial frontal cortices (preSMA, near dACC), and anterior thalamus/insula during 2-back vs. 0-back, and in the dACC, middle/posterior cingulate, bilateral somatomotor cortex, paracentral lobule, ventral precuneus, frontopolar cortex, and thalamus (pulvinar and habenula) during 0-back vs. 2-back (74). The bilateral dlPFC, PreMA, PA, and ACC/SMA are activated during the n-back task, with right hemisphere dominance (65).

Activity in the thalamus and subcortical regions is significantly correlated with working memory performance in the n-back task, with increased mean diffusivity in bilateral thalami significantly correlated with reduced performance and significant anticorrelation in thalamic nuclei projecting to prefrontal and posterior parietal cortices (91).

N-back task activations predict functional connectivity patterns and cognitive ability better than resting-state fMRI, with significant involvement of fronto-parietal and cerebellar regions. The 2-back vs. 0-back contrast predicts general cognitive ability (GCA) achieving a 0.50 correlation with GCA scores in 10-fold cross-validation, showing SMA, precuneus, and dlPFC activations, and anterior default mode network (aDMN) deactivations (101).

Training significantly impacts neural activation during the n-back task, with transient and sustained neural responses and significant activations in frontal and parietal cortex, modulated by the type and amount of retained information and task practice amount (78).

*OCD deficits:* OCD patients may show slower reaction times, higher error rates, and greater difficulty with increased cognitive loads (143, 144), indicating significant challenges in cognitive control and working memory during the task. But others find no significant differences (145, 146).

*Neural basis of deficits:* Some studies identified distinct neural activation patterns in patients with OCD during n-back tasks. Heinzel et al (62, 63) found reduced activation in the SMA, bilateral inferior parietal lobule (IPL), and dlPFC during high working memory load conditions (2-back and 3-back). Koch et al. (71) found significantly decreased activation in the dACC with increasing task demands during a verbal n-back task. Conversely, de Vries et al. (48) observed task-related *hyperactivation* in the left dlPFC and left precuneus with increased functional connectivity between frontal cortex and the bilateral amygdala.

### 3.8 The delay discounting task

*The task:*

“At the conceptual level, the term ‘delay discounting’ refers to a decline in subjective value with expected delay. A typical delay discounting experiment begins by presenting the participant with a choice between an immediate gain and a delayed gain of greater magnitude. The subjective value of the delayed gain is then calculated as the midpoint between the last amount of the immediate gain chosen over the delayed alternative and the last amount of the immediate gain rejected. This procedure, if repeated several times with varying magnitudes of delay, produces a number of points through which a ‘discounting curve’ can be drawn for each participant. The discounting curve can be assessed with two alternative classes of metric: (1) the Area Under the Curve (AUC), a simple empirical measure; or (2) the discount constant k, which assumes a specific underlying decay function. Both AUC and k are taken to reflect the underlying internal rate of delay discounting, and can be measured as state (i.e., showing within-person variability over time) or trait (i.e., showing within-person stability and between-person variability). The AUC for each individual participant is the total area of the trapezoids of his/her discounting curve and its obtained value will vary somewhat with the delay intervals chosen for testing. The smaller the AUC, the more the participant discounts the delayed gain. In the case of k, the discounting curve is usually modeled as a hyperbolic or exponential function, with recent research favoring the hyperbolic, given its better fit to the data. AUC and k are therefore thought to each adequately reflect the extent to which the participant discounts the delayed gains at the empirical level.” (58, pp. 49-50).

*Neurology:* The OFC, pivotal for representing reward values, integrates signals from striato-limbic regions (including CN, ACC, PCC, vmPFC, insula, and Parahippocampal Gyrus) processing reward properties with inputs from dlPFC cortex involved in temporal foresight and self-control. This integration facilitates a comprehensive evaluation of immediate versus delayed rewards, guiding decision-making towards more long-sighted outcomes (43, 61). The preference for delayed rewards correlates with heightened dlPFC activation, underscoring its role in moderating impulsivity through enhanced self-control and temporal planning (77). In conclusion, delay discounting tasks reveal a complex interplay between neural regions associated with valuation, self-control, and long-term planning. Higher rate of discounting is associated with a set of brain regions linked to valuation (e.g., Nucleus Accumbens, CN, Putamen, and ACC, PCC), while lower rate of discounting is associated with a set of structures known to be involved in long-term planning (dlPFC and OFC) (58).

*OCD deficits:* Some studies have found significantly higher delay discounting rates in OCD patients, indicating increased impulsivity, while other studies have reported no significant differences in delay discounting between OCD patients and healthy controls (See **Table 3**).

**Table 3:** Summary of DDT findings on OCD deficits.

Measure	Explanation of measure	Main findings	Study
The discounting rate (k)	A higher k value indicating a greater preference for immediate over delayed rewards.	OCD subjects exhibited a significantly higher delay discounting rate (k) in the DDT, indicating an increased level of choice impulsivity compared to healthy controls. These findings align closely with the clinical characteristics of OCD, which include a greater preference for avoiding risky situations, a marked inability to wait (which may provoke excess safety behaviors or compulsions), and a difficulty in stopping already initiated behaviors (or repetitions).	Sohn et al. (147)
The discounting rate (k)	A higher k value indicating a greater preference for immediate over delayed rewards.	OCD patients displayed significantly higher k-values than healthy controls.	Mavrogiorgou et al. (148)
The discounting rate (k)	A higher k value indicating a greater preference for immediate over delayed rewards.	An absence of performance differences in delay discounting tasks between OCD individuals and controls.	Steinglass et al. (149)
Discount factor	The discount factor is a numerical value that quantifies an individual’s preference in larger, delayed rewards over smaller, immediate ones, with a higher discount factor indicating a lesser degree of temporal discounting.	Examined the performance in an asymmetric discounting (intertemporal choice) task and found no differences between OCD subjects and controls.	Pinto et al. (150)
Discount factor	The discount factor is a numerical value that quantifies an individual’s preference in larger, delayed rewards over smaller, immediate ones, with a higher discount factor indicating a lesser degree of temporal discounting.	An absence of performance differences in delay discounting tasks between OCD individuals and controls.	Steinglass et al. (149)
AUC	A smaller AUC indicates steeper discounting rates, representing increased choice impulsivity.	An absence of performance differences in delay discounting tasks between OCD individuals and controls.	Carlisi et al. (41)
Number of choices for smaller immediate rewards	Quantified the preference for immediate rewards by counting the instances participants chose smaller, immediate rewards over larger, delayed ones in a trial-constraint condition.	An absence of performance differences in delay discounting tasks between OCD individuals and controls.	Vloet et al. (151)

*Neural basis of deficits:* The OFC and the caudate loops (including CN, Nucleus Accumbens, STN, GP, Thalamus, vmPFC, rACC, dACC, and vACC) are proposed to drive impulsivity and compulsive behavior, highlighting a critical link between neural dysfunctions and choice impulsivity in OCD (41, 46, 152). Functional magnetic resonance imaging (fMRI) studies have identified the OFC as a major cortical region of activity during these tasks, underscoring its role in reward valuation and decision-making processes (153). OCD patients exhibit neural under-activation in regions responsible for self-control and temporal foresight during temporal discounting tasks. Norman et al. (87) provided further insights by demonstrating that the OFC and the rostrolateral prefrontal cortex (rlPFC) were specifically under-activated in OCD patients during temporal discounting tasks, despite normal delay discounting rates (k values). This under-activation suggests a disorder-specific neural dysfunction in areas responsible for self-control and temporal foresight, including the right inferior frontal gyrus (IFG), dorsolateral prefrontal cortex (dlPFC), anterior insula, dorsal striatum, and bilateral cerebellum. Interestingly, only in OCD patients, this under-activation extended to regions involved in goal-directed reward evaluation and prospection, such as the right OFC and rlPFC, indicating a pronounced neural basis for choice impulsiveness in OCD (87). Conversely, the rlPFC, implicated in episodic prospection, planning, counterfactual thinking, and the representation of abstract, temporally extended goals (154–157), exhibited greater activation during immediate choices in OCD patients, in contrast to controls who showed greater activation during delayed choices (87).

## 4 Discussion

We adopted a structured, replicable framework focused on cross-task convergence of OCD-specific abnormalities as an objective metric of neural involvement. We identified brain regions showing convergent involvement across tasks exclusively from OCD cohorts (see **Table 2**). This multi-task approach provides the most comprehensive cross-domain synthesis to date, revealing patterns of neural convergence and divergence that single-task reviews cannot capture.

This data-driven approach distinguishes core from peripheral substrates for the first time on the basis of OCD individuals’ functional abnormalities, yielding a hierarchical model of cortico-striato-thalamo-cortical (CSTC) circuits that drive cognitive inflexibility in OCD and integrating this with classic circuit and modern network perspectives.

We integrated neuroimaging and neuromodulation data to strengthen inference. Convergent findings across modalities provide stronger evidence of neural involvement than any single method alone – leveraging the unique strengths of each method. We adopted a conservative synthesis strategy: regions were classified only when supported by multiple studies and, where possible, multiple modalities. This yielded a replicable distinction between core (generally shared) and peripheral (network-specific) substrates that is grounded in the data rather than *a priori* assumptions. This multilevel characterization provides a more unified framework for understanding the neural basis of cognitive inflexibility in OCD.

**Figure 3:**
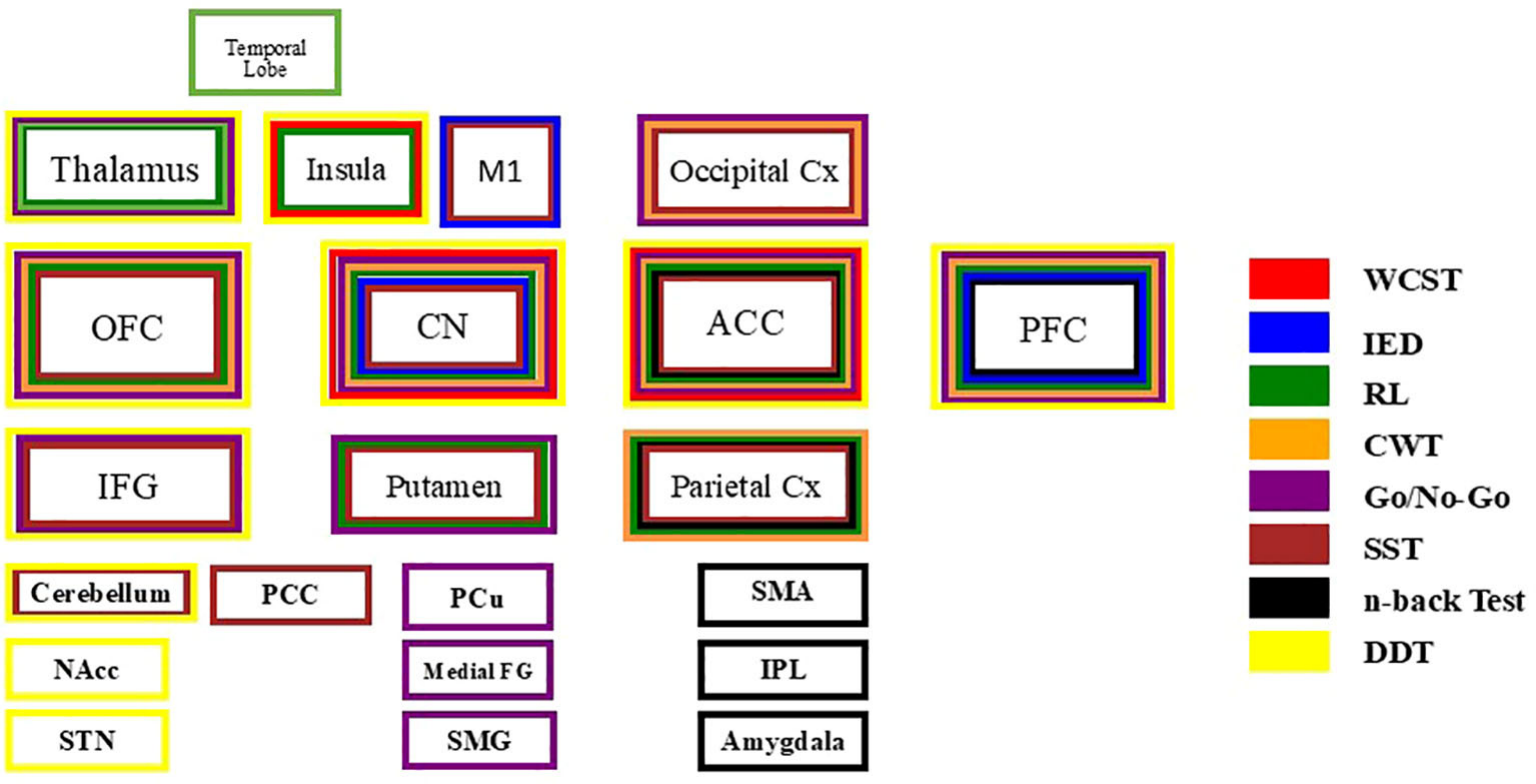
Neural basis of deficits in OCD patients by task. This is a visual summary of the data supplied in **Table 2**. Task involvement is coded by color. Structures are organized in relation to common task involvement. As discussed in the text OFC, CN, ACC, and PFC appear to form a core with largely overlapping task. Structures involved in fewer tasks are place more peripherally, upward or downward; with structures involved in only a single task at the outside involvement. For abbreviations see list at **Supplementary Table 1**. For colors see online version.

This model integrates data from various cognitive flexibility tasks and provides a comprehensive framework for understanding the neural mechanisms underlying OCD. We then discuss the apparent more task-specific involvement of other brain regions (§4.5–4.7). Our suggestion is that these are indirect, down-stream, effects of the core abnormalities in the CSTC and related circuits. The ‘core’ and ‘peripheral’ designations are primarily descriptive not dichotomous, based on the extent of engagement of OCD specific abnormalities across the tasks reviewed in the data in OCD individuals (as visualized in **Figure 3**, **Table 2**, OCD Task-Common/different columns), where core regions show broader involvement in multiple tasks, while peripheral regions are limited to fewer tasks. This distribution can be explained by a circuit-focused perspective, where the CSTC serves as the primary network, with salience and DMN acting as supporting systems through dynamic interactions. Recent neuroimaging studies support this view, emphasizing dynamic functional connectivity alterations in CSTC loops that extend to associated subcortical and cortical regions, contributing to cognitive inflexibility (158). For instance, abnormal dynamic functional connectivity in CSTC circuits correlates with symptom severity (159) and impaired task switching, while disruptions in salience-DMN interactions hinder network transitions necessary for flexible behavior (160).

In the following subsections and in **Figure 3**, based on the data in **Table 2**, we summarize the involvement of core brain regions exhibiting abnormal activity in OCD patients across the range of cognitive flexibility tasks: ACC (§4.1), CN (§4.2), OFC (§4.3), and PFC (§4.4). Any commonality across different cognitive flexibility tasks should provide a neurological basis for understanding cognitive inflexibility in OCD and the specific contribution of these brain regions to cognitive control. Importantly, despite their overlap none of these brain areas appears to be involved in the entire set of tasks. They, therefore, are likely to be components of interconnected networks.

### 4.1 Core brain regions – ACC

As can be seen from **Figure 3**, the ACC is implicated in almost all of the cognitive tasks commonly used to assess cognitive inflexibility (the IED is an exception). In the WCST, ACC dysfunction is associated with difficulties in adapting to new sorting rules after a shift, manifesting in perseverative errors (161). In Reversal Learning tasks, it is associated with poor error monitoring and adaptive control (30), potentially contributing to maladaptive, repetitive behavior (162). In the CWT Task, it may prolong reaction times and increase error rates to incongruent stimuli.

In tasks assessing inhibitory control, such as the Go/No-Go task and the SST, ACC abnormalities in OCD patients are linked to a reduced ability to inhibit prepotent or habitual responses (96, 163). Dysfunction in the ACC may contribute to the characteristic repetitive behaviors and difficulty in stopping compulsive actions seen in OCD. In the n-back Test, decreased ACC activation in OCD patients during higher working memory load conditions has been reported (62, 71). This indicates challenges in managing increased cognitive demands, suggesting that ACC dysfunction may affect working memory processes in OCD.

During the DDT, altered ACC activation has been observed in OCD patients, suggesting difficulties in integrating long-term consequences into decision-making processes (41). This dysfunction may correlate with the impulsivity and inability to delay gratification, exacerbating compulsive behaviors.

Functional neuroimaging studies have consistently implicated ACC dysfunction in OCD patients. Patients with OCD often exhibit hyperactivation or abnormal activity of the ACC during tasks requiring cognitive flexibility and inhibitory control (164). Dysfunction within the ACC disrupts conflict monitoring, error detection, and inhibitory control mechanisms, contributing to the cognitive inflexibility and repetitive behaviors characteristic of OCD.

Collectively, these findings underscore the ACC’s integral role in orchestrating cognitive flexibility and emotional regulation processes. Dysfunction within the ACC across these tasks disrupts critical mechanisms such as conflict monitoring, error detection, and inhibitory control, contributing significantly to the cognitive inflexibility and repetitive behaviors characteristic of OCD (164). ACC has long been seen to be involved in the processing of obsessions and linked to the processing of compulsion by CN (165). These differences in level of processing could account for ACC involvement in the n-back task but not IED with vice versa for CN. Abnormal activation in the ACC, has been associated with heightened error monitoring and conflict detection in OCD. This pattern of dysfunction may contribute to exacerbating obsessive thoughts by amplifying perceived discrepancies between intended and actual outcomes, potentially contributing to repeated checking or rumination (166, 167). Furthermore, this ACC dysfunction contributes to cognitive inflexibility in OCD by impairing conflict resolution and executive control, reducing the ability to shift focus or adapt behaviors in response to new information (168).

### 4.2 Core brain regions – CN

As can be seen from **Figure 3**, like ACC, CN is involved in the bulk of the tasks (n-back is the exception). In the IED and RL tasks, CN dysfunction has been linked to difficulties OCD patients have in adjusting their behavior in response to new feedback or rule changes, resulting in persistence in erroneous responses despite changing task demands (13). This reflects the CN’s role in cognitive flexibility and the ability to adapt to changing environmental contingencies – but with a more motor than cognitive orientation compared to ACC. In the WCST and the CWT, CN dysfunction is associated with difficulties in shifting cognitive sets and adapting to new rules, correlating with perseverative errors and reduced behavioral flexibility (108, 132).

In tasks assessing inhibitory control, such as the Go/No-Go and SST tasks (and, again, RL), altered CN activity or structure is linked to impaired response inhibition (80). This dysfunction manifests as an inability to suppress prepotent or habitual responses, associating with the observed deficits in inhibitory control in OCD patients (164).

Additionally, the DDT implicates CN dysfunction in impulsivity and impaired decision-making. Abnormal CN activity in OCD patients is linked to difficulty in delaying gratification and integrating long-term outcomes into decision-making processes (87).

Collectively, these findings underscore the CN’s role in regulating cognitive flexibility, response inhibition, and decision-making processes. Dysfunction within the CN contributes significantly to the cognitive inflexibility, habitual responses, and deficits in inhibitory control characteristic of OCD but at a more behavioral level than ACC. Dysfunction in the CN—a key component of the striatum within the dysfunctional CSTC circuit—has been associated with over-reliance on habitual (as opposed to goal-directed) system in OCD (169–171). This dysfunction contributes to compulsive actions by reinforcing rigid, maladaptive response patterns that resist modification, thereby reducing cognitive flexibility, particularly in contexts involving task shifts, feedback adaptation, or updating based on new information (13, 21, 172). Furthermore, this can perpetuate obsessive thoughts through sustained activation of striatal loops (e.g., CSTC circuits) that fail to appropriately update or devalue reward contingencies, leading to persistent, non-adaptive reinforcement of behaviors despite negative or neutral outcomes (173).

### 4.3 Core brain regions – PFC

**Figure 3** shows the PFC is involved in most of the cognitive tasks that assess cognitive flexibility and executive function (the WCST and SST are exceptions). In the IED and RL tasks, the PFC, especially the dlPFC, is critically involved in shifting attention, adjusting behavior, and updating task rules based on changing feedback. OCD patients often exhibit difficulties in these tasks due to frontostriatal dysfunction dysfunction, associating with impairments in cognitive flexibility and an inability to modify responses when confronted with new information or rules (13, 42).

In the n-back task, OCD patients display reduced dlPFC activity, manifesting as decreased working memory capacity (63) and slower information processing speed. The impairment in working memory also correlates with the broader executive dysfunction characteristic of OCD.

In tasks requiring response inhibition, such as the Go/No-Go task, the IFG, in conjunction with the dlPFC, plays a role in suppressing prepotent or inappropriate responses. This impairment in inhibitory control reflects broader challenges OCD patients face in regulating behavior and suppressing compulsive actions (174).

The CWT also implicates PFC dysfunction (55, 102). Abnormal PFC activity, particularly in the dlPFC and associated regions (64), is associated with prolonged reaction times and increased error rates when processing incongruent stimuli. This difficulty in managing interference and adapting behavior to task demands is an aspect of the cognitive inflexibility seen in OCD patients.

In the DDT, the vmPFC is crucial for integrating long-term consequences into decision-making processes. Reduced vmPFC activity in OCD patients is associated with the difficulty in weighing future outcomes, linking to the impulsivity and persistence of compulsive behaviors (87).

These findings emphasize the critical role of the PFC, particularly the dlPFC and vmPFC, in regulating cognitive flexibility, planning, working memory, and response inhibition. Dysfunction in these prefrontal regions disrupts the executive control necessary for adapting to changing environmental demands, making decisions, and suppressing inappropriate behaviors. This disruption is more biased towards working memory failure as a basis for continued inappropriate action than with the more generative problems of ACC and CN. This disruption, thus, plays a distinct significant role in the cognitive and behavioral symptoms of OCD, including the characteristic cognitive inflexibility and compulsivity. Dysfunction in the PFC (29), particularly the dlPFC, has been associated with diminished executive control in OCD, contributing to persistent obsessive thoughts through impaired top-down regulation of limbic responses (175) and reduced cognitive flexibility by hindering task switching and strategy updating (176). This may exacerbate compulsive behaviors by failing to inhibit habitual responses (177), as seen in altered frontoparietal connectivity (178).

### 4.4 Core brain regions – OFC

**Figure 3** shows that OFC, also, is involved in the bulk of the tasks. The exceptions are the WCST, IED and n-back test. In the RL and DDT, OFC dysfunction is associated with impaired evaluation of delayed rewards, which manifests in OCD patients as a preference for immediate, smaller rewards over larger, delayed rewards. This exhibits as impulsive decision-making, reflecting a failure to appropriately assess long-term outcomes (93). The inability to adjust reward values based on feedback further exacerbates maladaptive behaviors, reinforcing compulsivity.

In tasks assessing inhibitory control, such as the SST and Go/No-Go task, OFC dysfunction is linked to a diminished ability to inhibit impulsive or prepotent responses. This decreased inhibitory control results in challenges for OCD patients to regulate compulsive impulses and stop maladaptive behaviors (80). The OFC’s role in modulating response inhibition is crucial in maintaining behavioral flexibility, and its dysfunction contribute to the ability to halt compulsive actions. This action stopping aspect (demonstrated by the SST) distinguishes it from PFC, which shares with it more anticipatory response inhibition (demonstrated by the Go/No-Go task).

In the CWT, OFC abnormalities (102) are connected to difficulties in managing cognitive interference and hypersensitivity to errors or negative feedback. This sensitivity can cause excessive self-monitoring and an overemphasis on correcting mistakes, which are features of OCD-related compulsive behaviors. The inability to appropriately filter negative feedback may connect to the repetitive nature of compulsions and the inflexibility in decision-making seen in OCD patients.

These findings underscore the OFC’s role by mediating reward evaluation, inhibitory control, and feedback sensitivity. Dysfunction within the OFC disrupts these processes, contributing to the impulsive decision-making and compulsive behaviors characteristic of OCD. Aberrant activity in the OFC (179) is associated with dysfunctional reward and loss processing in OCD (180), promoting persistent obsessions via heightened sensitivity to threat cues (181) and impaired cognitive flexibility through inflexible outcome representations (11), which disrupts adaptive reversal learning and affective switching (182). Such alterations may maintain compulsions by dysregulating orbito-striatal pathways that impede the flexible updating of goal-directed behaviors (92).

### 4.5 Peripheral brain regions – putamen

Altered activity in the putamen has been linked to various cognitive and behavioral deficits observed in OCD, particularly in tasks requiring motor inhibition, and response control. However, it’s important to note that these changes may not represent a primary dysfunction within the putamen itself but could result from disrupted input from the CN.

In the RL task, where participants must adjust their behavior in response to changing reinforcement contingencies, altered putamen activity can impair the ability to suppress previously learned motor responses. This contributes to perseverative errors, making it difficult for OCD patients to shift strategies and adapt to new rules. As a result, patients may continue to engage in maladaptive, repetitive behaviors even when they are no longer appropriate or rewarding (92).

In the Go/No-Go and SST tasks, both of which assess response inhibition and the ability to suppress automatic or prepotent responses, the putamen is critically involved in controlling motor responses. Altered activity or structure in the putamen during these tasks may contribute to difficulties in inhibiting habitual or impulsive actions (80), contributing to the repetitive and compulsive behaviors characteristic of OCD.

In CSTC circuits, the putamen interacts with core regions like the CN and OFC to modulate habit formation (170); disruptions in CN-putamen connectivity may amplify compulsive behaviors by reinforcing rigid motor patterns (183), indirectly exacerbating cognitive inflexibility through impaired feedback integration in salience network dynamics (108).

### 4.6 Peripheral brain regions – thalamus

In the Go/No-Go task, altered thalamic activity (96) may disrupt the coordination between sensory input and motor output, resulting in difficulties in distinguishing between relevant and irrelevant stimuli that underlie the challenges OCD patients face in controlling compulsive behaviors.

In the RL task, the thalamus is involved in integrating feedback to facilitate appropriate behavioral adjustments. Abnormal thalamic activation in OCD patients (30) may impair their ability to modify responses based on new information, correlating with perseverative errors and maladaptive behavior.

In the CWT, altered thalamic activity may impair the integration of sensory information and the coordination of motor responses. This can be related with prolonged reaction times and reduced accuracy in resolving conflicts between incongruent stimuli.

During the DDT, abnormal activity in the thalamus (87) may disrupt the balance between immediate and delayed rewards, exacerbating the compulsive behaviors observed in OCD.

Collectively, these findings underscore the thalamus’s role in integrating sensory and motor information necessary for cognitive control. Altered thalamic activity can contribute to the deficits in cognitive flexibility, response inhibition, and decision-making seen in OCD patients. As a relay within CSTC loops, the thalamus interacts with core regions like the ACC and PFC; dysconnectivity from CN-thalamus pathways may hinder error signal transmission (184), worsening cognitive inflexibility by disrupting salience network attention shifts and DMN disengagement during tasks (185).

### 4.7 Peripheral brain regions – parietal cortex

In OCD patients, the clear dysfunction in the PFC is believed to impact the functioning of connected regions like the parietal cortex (164, 186). At least in relation to cognitive flexibility, the parietal cortex is a peripheral rather than core area.

Dysfunction in the PFC appears to lead to a range of parietal changes in OCD patients. In the n-back test, it may lead to reduced parietal activation, contributing to decreased working memory capacity and impaired ability to manage increasing cognitive load (63). In the SST, it can affect parietal cortex activity involved in sensory signals processing (49). In the RL task it impacts on the role of parietal cortex in attentional flexibility (42, 92, 94, 187), contributing to maladaptive, repetitive behaviors and difficulties in adjusting to new rules (92). In the CWT, it can influence parietal cortex activity related to sensory integration, reflecting challenges in managing attentional demands and sensory processing (164).

These findings emphasize the core role of the PFC in regulating working memory, inhibitory control, and attention, and how its dysfunction can impact the parietal cortex (186). As part of the frontoparietal network (188), the parietal cortex interacts with core PFC regions to support attention and integration (189); hypoactivation due to PFC dysconnectivity (175) may exacerbate cognitive inflexibility by impairing spatial attention and working memory updating (190), indirectly amplifying DMN rumination in OCD (191).

### 4.8 Neural circuits underlying OCD

To integrate the task-specific deficits reviewed above with broader neural mechanisms, we examine the interconnected circuits implicated in OCD that may underlie cognitive inflexibility.

#### 4.8.1 OCD and cortico-striato-thalamo-cortical circuits

The CSTC circuits involve neural pathways connecting cortical regions (including the OFC, ACC, and PFC) to subcortical structures such as the striatum (which includes the CN and putamen). There are return circuits via the thalamus forming loops that are crucial for executive functions (192). CSTC circuits are involved in goal-directed behavior, habitual behavior, and an arbitration system that allocates control between them (15, 193; for a simple overview, see 194). They have a critical role in OCD (1, 10, 192, 195).

In OCD, hyperactivity in the OFC and ACC, and increased activity in the CN and thalamus, appear to be involved in impaired goal-directed behavior and poor inhibitory control (164, 195). The imbalance between the “direct” (excitatory) and “indirect” (inhibitory) pathways within the CSTC circuits may result in the persistence of intrusive thoughts and repetitive behaviors characteristic of OCD (196, 197). Moreover, impaired functional connectivity between these regions disrupts the balance between goal-directed and habitual systems, causing excessive habitual responding and a struggle to shift to goal-directed actions (16, 198, 199). The arbitration mechanisms that should flexibly allocate control between these systems appear to be deficient (17).

Gillan and Robbins (16) emphasize that compulsive thoughts take over because they have become habitual, and the arbitration system fails to disengage from these habits. Fineberg et al. (152) suggest poor inhibitory control in goal-directed circuits contributes to the persistence of compulsive behaviors in OCD. Kwon et al. (200) highlight neuroimaging findings that support abnormalities in CSTC circuits in OCD patients.

#### 4.8.2 Dimensional and computational perspectives on goal-directed control in OCD

A complementary contemporary perspective conceptualizes cognitive inflexibility in OCD as an imbalance between goal-directed control, computationally indexed as model-based learning, and habitual responding. Within this framework, inflexible behavior may arise when behavior is insufficiently guided by flexible action–outcome evaluation and becomes relatively over-dependent on previously learned, automatic response tendencies (18, 193). In this sense, reduced goal-directed/model-based control is not opposed to habitual responding as a rival explanation, but may permit greater reliance on it.

Gillan et al. (18) demonstrated that deficits in goal-directed control, indexed computationally by reduced model-based learning, were most specifically associated with a transdiagnostic symptom dimension of compulsive behavior and intrusive thought, rather than with anxious-depression or social withdrawal.

These findings provide a mechanistic interpretation of how disruptions in goal-directed control may contribute to inflexible and compulsive behavior: reduced model-based learning would be expected to weaken flexible action–outcome evaluation and prospective control, thereby increasing reliance on overlearned or habitual responding (10, 17). Because **Table 2** was restricted *a priori* to the eight neuropsychological task domains reviewed in this manuscript, these dimensional and computational studies were not included in the core/peripheral cross-task counting procedure. Instead, they are discussed here as a convergent interpretive framework that strengthens the link between the CSTC abnormalities and the persistence of compulsive symptoms.

#### 4.8.3 OCD and other networks

The ACC, CN, OFC, and PFC together constitute the core of a complex interconnected set of neural networks (201). They are integral parts not only of the CSTC circuits but also the salience network, and the default mode network (DMN) – which have also been implicated in OCD (1, 191, 195).

*The salience network*, which includes the ACC and anterior insula, plays a crucial role in detecting and filtering salient stimuli and facilitating the switch between the DMN and executive control networks (202). Dysfunction in the salience network may contribute to difficulties in shifting attention and adapting to new tasks in OCD patients (108). Specifically, impaired functioning of the salience network can result in an inability to appropriately allocate attentional resources (203). For example, abnormalities in the ACC within the salience network can disrupt error detection and conflict monitoring, essential functions for adjusting behavior in response to changing environmental demands (204).

*Similarly, the DMN*, involving regions such as the medial PFC and posterior cingulate cortex, is associated with self-referential thinking and mind-wandering (205, 206). Alterations in the DMN have been linked to increased self-focused rumination and intrusive thoughts in OCD patients (191). This excessive internal focus can interfere with the ability to engage in goal-directed behavior and to flexibly shift attention to external tasks (191). The inability to disengage from internally driven obsessive thoughts may prevent OCD patients from adapting their behaviors in response to external feedback or changing circumstances.

*The frontoparietal network (FPN)*, encompassing regions like the dlPFC and inferior parietal cortex, supports executive functions such as working memory and cognitive control and interacts with CSTC circuits in OCD (207).

#### 4.8.4 OCD and network dysfunction

OCD appears to involve large-scale network dysconnectivity. Gürsel et al. (207) found that OCD is characterized by hypoconnectivity and general dysconnectivity within and between the FPN, salience network, and default mode network (DMN), supporting the application of the triple network model to highlight disturbed interplay among them. Recent dynamic functional connectivity studies have extended these findings (190), revealing disrupted entropy and temporal variability in these networks, including reduced entropy in the dorsal attention network and increased entropy in the DMN, caudate nucleus, and striatal regions, which contribute to inefficient network switching and persistent habits; and hindering shifts from internal self-referential processing (DMN) to goal-directed cognitive control (FPN) in response to external demands (190, 208).

Such dysconnectivity supports models emphasizing fronto-striatal circuit abnormalities central to OCD pathophysiology and is associated with patients’ inability to inhibit obsessive thoughts and compulsive actions (207, 209). Integrating these models indicates that frontoparietal network abnormalities are pivotal to OCD’s core symptoms, providing key insights into underlying neural mechanisms (207, 210). These findings offer important insights into the neural mechanisms underlying OCD, suggesting that aberrant connectivity in large-scale brain networks is key to understanding the disorder’s core symptoms (207).

The CSTC circuits involve loops between cortical areas (such as the OFC, ACC, and PFC) and subcortical structures (like the CN, putamen and thalamus); whereas the frontoparietal network includes regions in the frontal and parietal cortex (such as the dlPFC and inferior parietal cortex). While anatomically distinct, they interact and overlap in their functions. Abnormalities in both contribute to the symptoms observed in OCD resulting in both cognitive inflexibility and a failure of goal-direct planning (13).

### 4.9 Interconnected neural networks and cognitive flexibility

What we have termed the core regions for cognitive inflexibility—including the ACC, CN, OFC, and PFC—are consistently implicated across the bulk of the cognitive flexibility tasks we have reviewed on the basis of OCD individuals’ data (**Table 2**). As a set they appear to have a central role in the cognitive inflexibility observed in OCD (164, 195). They also, as a set but not individually, are involved in a range of neural networks implicated in OCD. As shown in **Table 4**, they are all involved in the CSTC circuits; ACC is involved in the salience network; and PFC is involved in the DMN.

**Table 4:** Involvement of brain regions in neural networks linked to OCD.

Brain region	CSTC circuits	Salience network	DMN
OFC	✔ 164, 192, 195, 211		
ACC	✔ 164, 192, 195, 211	✔ 202, 204, 212, 213	
PFC	✔ 164, 192, 211		✔ 214–217
CN	✔ 164, 192, 195, 211		
Putamen	✔ 164, 192, 195, 211		
GP	✔ 164, 192, 195, 211		
Thalamus	✔ 164, 192, 195, 211		
AI		✔ 202, 204, 212, 213	
PCC and Precuneus			✔ 214–217
Parietal Cortex (IPL and Angular Gyrus)			✔ 214–217
Hippocampus and Parahippocampal Gyrus			✔ 214, 215, 217

Their multi-network involvement suggests they play a central role in the cognitive inflexibility observed in OCD. An important question arises: Does cognitive inflexibility in OCD involve all the neural networks associated with the disorder? Or, is it primarily driven by dysfunction in one or more specific core networks, such as the CSTC circuits? Understanding this is crucial for unraveling the neural basis of OCD.

We will argue that, cognitive inflexibility in OCD predominantly involves the core CSTC circuits, but it is also mediated by the other neural networks to varying degrees (10, 13). Specifically, the ACC is part of both the CSTC circuits and the salience network – with the insula showing some involvement in cognitive inflexibility. Likewise, the PFC is involved in both the CSTC and DMN – with, e.g., parietal cortex showing some involvement in cognitive inflexibility. This suggests that disruptions in the core regions could affect multiple networks (202, 204). That said, these core areas seem to be central not only for cognitive inflexibility but also for other dysfunctions in OCD, highlighting their critical role in the disorder’s neuropathology (10). This centrality may be due to their involvement in fundamental cognitive processes such as executive function, decision-making, and inhibitory control (3, 192). Dysfunction in these areas disrupts the balance between goal-directed and habitual behaviors, contributing to the characteristic symptoms of OCD (193, 199).

In that sense, while cognitive inflexibility involves dysfunction related to multiple networks and results from their disconnection, cognitive flexibility may be viewed as more coherent – being the emergent result of their integrated interactions.

### 4.10 CSTC circuits as the core network

Cognitive inflexibility appears to arise from dysfunction of a set of core areas embedded in the CSTC circuits. This results in the inability to switch between behaviors based on changing environmental stimuli (16). However, the CSTC includes areas that we see as less generally involved in cognitive inflexibility. Abnormal activity in the OFC, ACC and CN, can combine with that in putamen and globus pallidus, to generate persistence of intrusive thoughts and compulsive behaviors (164, 195). Likewise, the thalamus, acting as a relay station within the CSTC circuits, can occasionally play a role in modulating these processes (218). This hyperactivity (3) contributes to impaired inhibitory control—a critical function in cognitive flexibility—due to imbalances in these regions (164). These imbalances disrupt the arbitration between goal-directed and habitual actions, contributing to the compulsive behaviors and cognitive inflexibility characteristic of OCD (17, 193). Thus, the CSTC circuits appear to contain the core network driving the neural deficits underlying cognitive inflexibility in OCD (see also 1). Recent neuroimaging studies have implicated imbalances in CSTC loops (210, 219), where altered functional connectivity between OFC and striatum (220, 221) perpetuates habit dominance (220), reducing flexibility in adapting to new contingencies and fostering compulsive rituals.

### 4.11 Salience network as a supporting network

The salience network, which involves ACC and the anterior insula, is responsible for detecting and filtering salient stimuli (204), and for facilitating the switch of activation between the DMN and executive control networks (202).

Although the salience network is not directly responsible for executive functions like cognitive flexibility, its role in attention allocation and in regulating shifts between different brain networks plays a role in cognitive inflexibility in OCD. Dysfunction in the salience network exacerbates difficulties in attentional control and the detection of task-relevant stimuli, contributing to the broader impairment in cognitive flexibility (203). Therefore, while the salience network is not a primary network generating cognitive inflexibility, the ACC’s involvement in both the CSTC circuits and the salience network suggests that dysfunction in this region might have widespread effects on cognitive inflexibility, it possibly serves a supporting role by facilitating the switch between the DMN and other networks during task performance (202). Disruptions in salience network dynamics, such as delayed switching from DMN to task-positive states (222), may amplify cognitive inflexibility in OCD (223) by prolonging error-related signaling (224), linking to obsessive rumination and reduced adaptability in tasks like reversal learning.

### 4.12 DMN as a peripheral network

The DMN is primarily associated with self-referential thinking, mind-wandering, and rumination (205, 206). OCD patients frequently exhibit hyperactivity in the DMN, which correlates with increased rumination and obsessive thoughts (191). This excessive internal focus can interfere with task engagement and cognitive flexibility by preventing efficient shifts to task-positive networks, such as the FPN.

However, the DMN’s contribution to cognitive inflexibility is more indirect. Rather than driving cognitive inflexibility, its dysfunction supports internal distractions that further complicate task performance and cognitive flexibility (202). As shown in **Table 4**, the DMN includes regions such as the PFC, PCC, precuneus (excluding area 7m), parietal cortex (including the IPL and angular gyrus), hippocampus, and parahippocampal gyrus (214). Hyperactivity in these regions might contribute to the excessive internal focus observed in OCD patients (191). Thus, we consider the DMN to be a peripheral network in the neural deficits underlying cognitive inflexibility in OCD. Connectivity alterations within the DMN in OCD hinders disengagement from self-referential processing (225), indirectly impairing cognitive flexibility (226) by sustaining obsessive thoughts during task performance (227), potentially linked to dysconnectivity with salience and executive networks, including aberrant fronto-striatal interactions within CSTC circuits (207).

### 4.13 Peripheral regions: putamen, thalamus and parietal cortex

In contrast, regions like the putamen, thalamus and parietal cortex may be considered ‘peripheral’ because their impairments are likely downstream effects of abnormalities in the core CSTC circuits. Although the putamen and thalamus are integral to CSTC, their limited task-specific abnormalities suggest downstream effects from core hubs like CN and OFC (175, 184, 228). While these peripheral regions appear to play important roles in specific tasks – their involvement across tasks is quite limited and appears to be less direct than that of the core areas.

They are key brain areas for the normal execution of the tasks and so task disruption via the core network would affect their activity. Impairment in these areas may interact with core circuit dysfunctions within the CSTC circuits, potentially exacerbating cognitive or behavioral difficulties. For instance, the thalamus acts as a relay station, influencing cognitive processes indirectly by modulating information flow between cortical and subcortical regions (218). Thalamic impairment has been suggested to contribute to delays or errors in information transmission, potentially disrupting the smooth progression of cognitive processes but is not consistently implicated across all cognitive flexibility tasks in OCD (164).

Given that the thalamus is part of the CSTC circuits (**Table 4**) but does not show the same pattern as the core areas in relation to cognitive inflexibility, its apparent impairment might be correlated with selective disruptions in CSTC pathways rather than a primary cause (195). This suggests that while the thalamus is important for overall cognitive processing, its role in cognitive inflexibility in OCD is secondary to that of the core CSTC regions. Altered activity in the thalamus (229) may result from disrupted input from the CN, affecting its role in information relay (230).

Welter et al. (230) demonstrated that impairment in the STN, which is closely connected to the thalamus, has been linked to OCD symptom severity and can predict the efficacy of high-frequency stimulation treatments like deep brain stimulation (DBS). Abnormal neuronal activity in the STN may disrupt its normal modulatory role within the basal ganglia circuitry, potentially affecting the balance of neural activity in the CSTC circuits and contributing to cognitive inflexibility and compulsive behaviors (229, 230). This indicates that while the thalamus and related structures are involved, they may not be the primary source of cognitive inflexibility but rather part of a network affected by core circuit dysfunctions.

Similarly, abnormal activity in the parietal cortex has been associated with difficulties in attention allocation, which may contribute to challenges in processing multiple tasks or when rapid responses are required, but may not be consistent impairment across tasks assessing cognitive flexibility in OCD. Its involvement may be more task-specific and less central to the core mechanisms underlying cognitive inflexibility.

As the parietal cortex, including the IPL and angular gyrus, is part of the DMN (214; **Table 4**), its impairment may contribute to the excessive internal focus and attentional difficulties observed in OCD. For example, parietal cortex impairment may stem from impaired connectivity with the PFC, influencing attentional processes indirectly (42, 49, 92, 94). de Vries et al (48) found that OCD patients exhibit compensatory activity in the frontoparietal network during working memory tasks, suggesting that parietal involvement might be a secondary effect of prefrontal abnormalities.

Likewise, the putamen is involved in motor control and habit formation (231); and so impairment in the putamen might contribute to the habitual behaviors seen in OCD. But its role is secondary to that of the core CSTC regions (164) with only limited involvement across the battery of cognitive flexibility tests. Recent studies have emphasized dynamic network interactions, where disruptions in CSTC-salience-DMN connectivity contribute to cognitive inflexibility (226) through impaired switching and sustained hypervigilance (223), as seen in altered functional connectivity patterns (226, 232).

### 4.14 Conclusions

In summary, cognitive inflexibility in OCD appears to result from functional abnormalities in a core set of structures (ACC, CN, OFC, PFC) embedded within multiple interconnected neural networks (**Table 4**). The CSTC network appears to play a central role, with additional contributions from the salience network and the DMN. Dysfunctions in the core brain regions results in impaired integration and processing of information, and is compounded by indirect effects on what we have termed ‘peripheral’ brain regions (putamen, thalamus, parietal cortex) that are parts of the same networks. While we do not see the peripheral areas as part of the core OCD circuitry in relation to cognitive inflexibility, there is some evidence suggesting their involvement, albeit less central, to the OCD’s primary mechanisms as downstream effects of core CSTC disruptions (195). Targeting the core regions (ACC, CN, OFC, PFC) may theoretically be more effective in alleviating cognitive inflexibility and related symptoms; whereas addressing peripheral regions may have limited impact unless core dysfunctions are also addressed (10, 13).

These findings have direct implications for neuromodulation in OCD, suggesting that targeting core regions within the CSTC circuits may yield greater therapeutic efficacy in alleviating cognitive inflexibility compared to peripheral areas. For instance, DBS of the ventral capsule/ventral striatum, which modulates CSTC loops including the CN and OFC (233, 234), has been shown to normalize hyperconnectivity (235) and improve cognitive inflexibility in refractory OCD patients (234), with response rates up to 60% in long-term follow-ups (236). Similarly, TMS (specifically intermittent theta burst stimulation) directed at the dlPFC has demonstrated enhancements in cognitive flexibility, as measured by reduced perseverative errors on set-shifting tasks (237). Recent closed-loop neuromodulation approaches, which can inform adaptation of stimulation based on real-time neural periodicity in key nodes of the CSTC circuits, such as the ventral striatum interconnected with the OFC and ACC, have been associated with clinical improvements in cognitive inflexibility-related symptoms, offering a personalized strategy to restore network balance (238). However, interventions addressing downstream regions alone, such as thalamic DBS, have shown limited efficacy in some studies, potentially due to secondary alterations arising from disrupted upstream CSTC input (239); concurrent mitigation of core upstream dysfunction may improve outcomes, though further research is needed to confirm this.

Cognitive inflexibility, in turn, may underlie the manifestation of compulsive behaviors, although this is likely driven by a combination of altered neural circuits and broader neurocognitive factors. Interestingly, greater cognitive flexibility may be associated with less severe OCD symptoms. The extent to which patients engage in perseverative behavior provides an indication of their level of cognitive inflexibility (240, 241). Understanding cognitive inflexibility is crucial in unraveling the roots of OCD symptoms and developing effective treatment strategies. Studies on cognitive flexibility help explain OCD’s core symptoms, such as repetitive thoughts and resistance to change (120, 242), by linking them to learning and neural plasticity. This connection underscores the importance of cognitive flexibility in the context of OCD.

Future research should address key unanswered questions to further elucidate neural mechanisms of cognitive inflexibility in OCD, including the dynamic interactions among CSTC, salience, and DMN networks during task switching. Longitudinal studies incorporating multi-modal imaging combining fMRI with EEG, could track cognitive inflexibility progression from prodromal stages to identify early biomarkers for intervention. Additionally, randomized controlled trials testing adaptive neuromodulation paradigms, such as closed-loop DBS or intensified TMS, should evaluate their potential specificity in remediating cognitive inflexibility, with pre-post assessments using standardized tasks like the Intra/Extra-Dimensional Set Shift. Integrating machine learning with connectomics may enable personalized mapping of aberrant circuits, addressing heterogeneity in OCD subtypes and comorbid conditions (243, 244). Ultimately, these directions could bridge neurobiological insights with clinical translation, fostering novel therapies that enhance cognitive flexibility and reduce symptom burden (245, 246).

## Glossary

ACC: Anterior Cingulate Cortex
aDMN: Anterior Default Mode Network
BA: Brodmann Area
CGT: Cambridge Gambling Task
CN: Caudate nucleus
CSTC: cortico-striato-thalamo-cortical
CWT: Color and Word Test
d: dorsal
dl: Dorsolateral
DBS: Deep Brain Stimulation
DDT: Delay Discounting Task
EDS: Extradimensional Set-Shifting
FRN: Feedback-Related Negativity
GAD: Generalized Anxiety Disorder
GP: Globus Pallidus
IED: Intra/Extra-Dimensional Set Shift Task
IFG: Inferior Frontal Gyrus
IPL: Inferior Parietal Lobule
l: lateral
M1: Primary Motor Cortex
m: Medial
MFG: Middle Frontal Gyrus
OCD: Obsessive-Compulsive Disorder
OFC: Orbitofrontal Cortex
OFG: Orbitofrontal Gyrus
PC: Parietal Cortex
PCC: Posterior Cingulate Cortex
PCu: Precuneus
PFC: Prefrontal Cortex
PreMA: Premotor Areas
pre-SMA: Pre-Supplementary Motor Area
rdl: Right Dorsolateral
rl: Rostrolateral
SAD: Social Anxiety Disorder
SMA: Supplementary Motor Area
SMG: Supramarginal Gyrus
SST: Stop Signal Task
SSRIs: Selective Serotonin Reuptake Inhibitors
STN: Subthalamic Nucleus
TMS: Transcranial Magnetic Stimulation
vl: Ventrolateral
vm: Ventromedial
WCST: Wisconsin Card Sorting Test
WM: Working Memory
