# Supplementary material for: Neural mechanisms underlying cognitive inflexibility in obsessive-compulsive disorder: a review
Source: Front Psychiatry. 2026 Jul 20;17:1795436. doi: 10.3389/fpsyt.2026.1795436 (PMC13430122; doi:10.3389/fpsyt.2026.1795436)
Supplement: Supplementary file 1 [file SupplementaryFile1.pdf]

Supplementary Table S1 List of Abbreviations

ACC: Anterior Cingulate Cortex
aDMN: Anterior Default Mode Network
BA: Brodmann Area
CGT: Cambridge Gambling Task
CN: Caudate nucleus
CSTC: cortico-striato-thalamo-cortical
CWT: Color and Word Test
d: dorsal
dl: Dorsolateral
DBS: Deep Brain Stimulation
DDT: Delay Discounting Task
EDS: Extradimensional Set-Shifting
FRN: Feedback-Related Negativity
GAD: Generalized Anxiety Disorder
GP: Globus Pallidus
IED: Intra/Extra-Dimensional Set Shift Task
IFG: Inferior Frontal Gyrus
IPL: Inferior Parietal Lobule
l: lateral
M1: Primary Motor Cortex
m: Medial
MFG: Middle Frontal Gyrus
OCD: Obsessive-Compulsive Disorder
OFC: Orbitofrontal Cortex
OFG: Orbitofrontal Gyrus
PC: Parietal Cortex
PCC: Posterior Cingulate Cortex
PCu: Precuneus
PFC: Prefrontal Cortex
PreMA: Premotor Areas
pre-SMA: Pre-Supplementary Motor Area
rdl: Right Dorsolateral
rl: Rostrolateral
SAD: Social Anxiety Disorder
SMA: Supplementary Motor Area
SMG: Supramarginal Gyrus
SST: Stop Signal Task
SSRIs: Selective Serotonin Reuptake Inhibitors
STN: Subthalamic Nucleus
TMS: Transcranial Magnetic Stimulation
vl: Ventrolateral
vm: Ventromedial
WCST: Wisconsin Card Sorting Test
WM: Working Memory

Supplementary Table S2 Common Brain Regions and Differential Neural Basis of Various Tasks (for abbreviations see list of abbreviations in Supplementary Table S1).

Task	Task-Common	Task-Different
**WCST**	PFC [45, 57, 56, 6, 55]	
**IED**	laPFC [68], mPFC [80], ACC [80], rdlPFC (BA 10 and 9/46) [68], Insula [80].	Ventral Tegmental Area [80], Periaqueductal Grey [80], Nucleus Accumbens [80], amSTN [82].
**RL**	OFC [1, 14, 65, 66], aOFC [1], dlPFC [1, 14, 65, 66, 67].	CN, putamen [1], Hippocampus [88].
**CWT**	ACC [10, 42, 25, 54], dlPFC [10, 42, 25, 54].	
**Go/ No-Go**	IFG[11], ACC [11], MFG[11], lOFC [52].	
**SST**	ACC [3, 8, 29, 12, 18, 30, 39, 46, 41, 61, 90, 91], MFG [9, 8, 29, 12, 20, 46, 49, 41, 63, 90, 91], IFG [3, 8, 12, 9, 20, 30, 41, 72, 18, 46, 49, 87, 61, 63, 29, 40, 70, 90, 4, 91]; Insula [3, 8, 9, 12, 18, 20, 39, 41, 46, 49, 70, 72, 90]	pre-SMA [3, 8, 20, 77, 78, 29, 40, 41, 46, 49, 9, 39, 12, 18, 61, 30, 70, 72], Parietal Cortex [3, 8, 78, 8, 9, 12, 18, 46, 49, 87, 29, 20, 39, 61, 63, 70, 90, 91], CN[18, 8, 12, 29, 46, 90], putamen[3, 18, 29, 46, 49, 63, 90]; nucleus accumbens, pallidum[3, 12, 18, 39, 46, 90]; STN[3, 41, 18, 72]; substantia nigra [39]; Basal ganglia (unspecified parts) [3, 8, 29, 90, 18, 39, 41, 46, 49, 61, 72, 87, 63, 91], Visual Areas [9, 8, 12, 20, 18, 29, 33, 39, 41, 46, 70, 90, 91], Cerebellum [20, 39, 41, 49, 61, 70, 90].
**n-back Test**	PFC [48, 38, 51, 64, 74, 79, 47, 23, 24, 76], MFG [23, 38, 47, 76], vlPFC [24], dlPFC	SMA [23, 38, 48, 76, 47, 74], PreMA [47, 48, 23, 24, 38, 64, 76],

	[76, 24, 74, 38], ACC [76, 79, 47, 38], IFG [24, 38, 47, 76], Insula [38, 47, 76, 79].	Parietal Cortex [48, 23, 24, 38, 64, 74, 76, 79, 51, 47], Premotor Cortex [64, 47, 76, 23, 24], M1 [24, 76], Somatomotor Cortex [24, 47, 76], Paracentral Lobule [47, 76], Visual Association Areas (Lingual and Fusiform Gyri) [23, 24, 79, 20], Occipital Cortex [23, 38, 24, 64], Thalami [38, 47, 64, 76], PCC [38, 47, 74, 76], aDMN [74], Cerebellum [23, 47, 76].
**DDT**	OFC[31, 34, 50], dlPFC [16, 31, 34, 50], vmPFC [16, 34], ACC [31, 16], Insula [34, 16].	Parahippocampal Gyrus [50], Nucleus Accumbens [31], PCC [31, 16, 34, 50], CN [16, 31, 34], Putamen [31, 16, 34].

Note:

The general tasks' common brain regions and differential neural basis (Task-Common and Task-Different) have been moved to Supplementary Table S1 for reference only and were not used for the core/peripheral distinction.

List of citations:

1. Ahmari & Rauch, 2022
2. Apergis-Schoute et al., 2017
3. Aron & Poldrack, 2006
4. Aron et al., 2003
5. Balzus et al., 2023
6. Bechara et al., 1996
7. Becker et al., 2023
8. Boehler et al., 2010
9. Boehler et al., 2011
10. Botvinick et al., 2001
11. Borgwardt et al., 2008
12. Cai et al., 2014
13. Carlisi et al., 2017
14. Chamberlain et al., 2008
15. Chamberlain et al., 2021
16. Christakou et al., 2011
17. Churchwell et al., 2009
18. Coxon et al., 2016
19. Dalley et al., 2011
20. Damiani et al., 2024
21. De Vries et al., 2014
22. De Wit et al., 2012

23. Dong et al., 2016
24. Dores et al., 2017
25. Egner & Hirsch, 2005
26. Endrass et al., 2013
27. Hajcak & Simons, 2002
28. Fajnerova et al., 2020
29. Fauth-Bühler et al., 2012
30. Floden & Stuss, 2006
31. Frost & McNaughton, 2017
32. Hampshire et al., 2020
33. Hanes et al., 1998
34. Hare et al., 2014
35. Heinzel et al., 2018
36. Heinzel et al., 2021
37. Hough et al., 2016
38. Huang et al., 2016
39. Ide & Li, 2011
40. Jha et al., 2015
41. Karoly et al., 2014
42. Kerns et al., 2004
43. Kıvırcık et al., 2003
44. Koch et al., 2012
45. Konishi et al., 1998
46. Li et al., 2006
47. Li et al., 2021
48. Liao et al., 2012
49. Marakshina et al., 2017
50. McClure et al., 2004
51. McEvoy et al., 1998
52. McNaughton et al., 2016
53. Menzies et al., 2007
54. Milham et al., 2001
55. Milner, 1963
56. Nagano-Saito et al., 2008
57. Nakahara et al., 2002
58. Narayanaswamy et al., 2019
59. Nishat et al., 2021
60. Norman et al., 2017
61. Obeso et al., 2013
62. Page et al., 2009
63. Pas et al., 2019
64. Piras et al., 2010
65. Remijnse et al., 2006
66. Remijnse et al., 2009
67. Remijnse et al., 2013
68. Rogers et al., 2000
69. Roth et al., 2007
70. Roxburgh et al., 2022
71. Schlösser et al., 2010
72. Sebastian et al., 2017
73. Snyder et al., 2015
74. Sripada et al., 2020
75. Stein & Ludik, 2000
76. Sweet et al., 2006
77. Tabu et al., 2011
78. Tabu et al., 2012
79. Tang et al., 2010
80. Tolomeo et al., 2018
81. Tomiyama et al., 2019
82. Tyagi et al., 2019
83. Valerius et al., 2008
84. Vaghi et al., 2017
85. Veale et al., 1996
86. Verfaillie et al., 2016
87. Vink et al., 2015
88. Vilà-Balló et al., 2017
89. Voon et al., 2017
90. White et al., 2014
91. Zheng et al., 2008

Supplementary Table S3 PRISMA Checklist

Section and Topic	Item #	Status	Location/Justification
**TITLE**	1	Partially reported	It is narrative review; but search strategy is systematic (Methods 2.1).
**ABSTRACT**	2	Partially reported	Abstract
**INTRODUCTION-RATIONALE**	3	Reported	Introduction (Section 1)
**INTRODUCTION-OBJECTIVES**	4	Reported	Introduction (Section 1)
**METHODS-ELIGIBILITY CRITERIA**	5	Reported	Methods 2.2
**METHODS-INFORMATION SOURCES**	6	Reported	Methods 2.1
**METHODS-SEARCH STRATEGY**	7	Reported	Methods 2.1 + Table 1
**METHODS-SELECTION PROCESS**	8	Partially reported	Methods 2.3 + Figure 1 (PRISMA flow diagram)
**METHODS-DATA COLLECTION PROCESS**	9	Reported	Methods 2.4
**METHODS-DATA ITEMS**	10a/10b	Reported	Methods 2.4
**METHODS-RISK OF BIAS ASSESSMENT**	11	N/A	Narrative review; no formal risk-of-bias tool was applied
**METHODS-EFFECT MEASURES**	12	N/A	Narrative review
**METHODS-SYNTHESIS METHODS**	13a–f	Reported for 13a–c; N/A for 13d–f	Methods 2.4 (narrative synthesis only; no statistical synthesis)
**METHODS-REPORTING BIAS ASSESSMENT**	14	N/A	Narrative review; no statistical synthesis or meta-analysis performed.
**METHODS-CERTAINTY ASSESSMENT**	15	N/A	Narrative review; no formal certainty assessment (e.g., GRADE) was used.
**RESULTS-STUDY SELECTION**	16a/16b	Reported	Methods 2.3 + Figure 1 (PRISMA flow diagram)
**RESULTS-STUDY CHARACTERISTICS**	17	Reported	Results (Section 3) + Table 2
**RESULTS-RISK OF BIAS IN STUDIES**	18	N/A	Narrative review
**RESULTS-RESULTS OF INDIVIDUAL STUDIES**	19	Reported	Results (Section 3) + Table 2
**RESULTS-RESULTS OF SYNTHESES**	20a–d	Reported / N/A for 20b–d	Results (Section 3) + Table 2 (narrative synthesis only)
**RESULTS-REPORTING BIASES**	21	N/A	Narrative review
**RESULTS-CERTAINTY OF EVIDENCE**	22	N/A	Narrative review
**DISCUSSION-INTERPRETATION**	23a	Reported	Discussion (Section 4) + Figure 3
**DISCUSSION-LIMITATIONS**	23b/23c	Reported	Discussion (Section 4)
**DISCUSSION-IMPLICATIONS**	23d	Reported	Discussion (Section 4)
